# From Theory to Practice: Advanced Nonlinear Optics and Multicolor, Tunable Fluorescence of Acedan Dyes

**DOI:** 10.1021/acs.jpcb.4c07533

**Published:** 2025-02-25

**Authors:** Alina Szukalska, Anna Grabarz, Bartłomiej Potaniec, Maria Zdończyk, Anna Popczyk, Karolina Waszkowska, Houda El Karout, Joanna Cybińska, Bouchta Sahraoui, Jarosław Myśliwiec

**Affiliations:** 1 Soft Matter Optics Group, Wrocław University of Science and Technology, Wyb. Wyspiańskiego 27, 50-370 Wrocław, Poland; 2 Department of Physical and Theoretical Chemistry, Faculty of Natural Sciences, Comenius University, Ilkovičova 6, 84215 Bratislava, Slovakia; 3 Łukasiewicz Research Network − PORT Polish Center for Technology Development, ul. Stabłowicka 147, 54-066 Wrocław, Poland; 4 University of Wrocław, Faculty of Chemistry, ul. F. Joliot-Curie 14, 50-383 Wrocław, Poland; 5 Humboldt Centre for Nano- and Biophotonics, Department of Chemistry, University of Cologne, 50923 Cologne, Germany; 6 Univ Angers, LPHIA, SFR MATRIX, F-49000 Angers, France; 7 Univ Angers, CNRS, MOLTECH-ANJOU, SFR MATRIX, F-49000 Angers, France

## Abstract

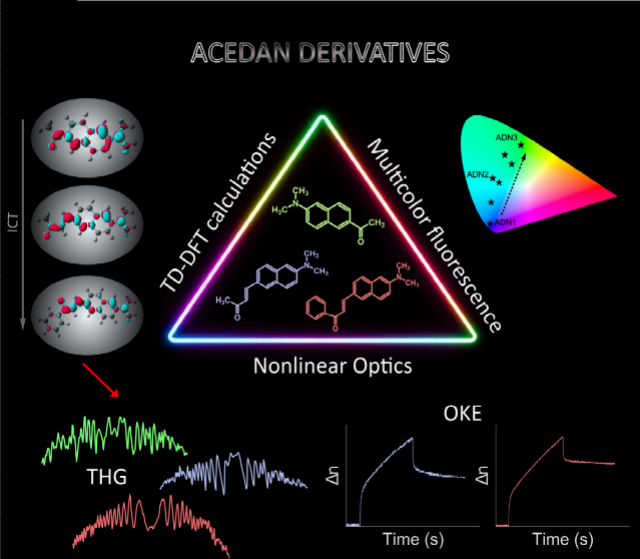

Acedan (ADN) and its derivatives are versatile dyes known for their donor–acceptor properties that can be fine-tuned for numerous spectroscopic applications. Currently, they are widely used as fluorescent probes for labeling biomolecules and cellular organelles. This study examines the newly discovered multifunctionality of three ADN chromophores, amplifying their application perspectives. We employ TD-DFT methods to guide, discuss, and support experimental research. Furthermore, by utilizing nonlinear optical (NLO) techniques such as the Maker fringes method to evaluate third harmonic generation (THG) and all-optical switching (optical Kerr effect, OKE), we show that ADN derivatives exhibit remarkable NLO properties. Specifically, in THG experiments, ADN1, ADN2, and ADN3 reveal signals approximately 2.5, 2.0, and 12.0 times stronger, respectively, than the reference material (silica). Additionally, the OKE experiment confirms ADNs’ photoinduced birefringence. Examined acedanes can also exhibit polychromatic fluorescence and energy transfer between individual components in two- and three-dye arrangements. Consequently, this comprehensive study offers valuable insights for applications, such as light-emitting diodes, sensors, projectors, and displays.

## 1 Introduction

Organic materials have become highly valuable in optoelectronics due to their intriguing linear and nonlinear optical (NLO) properties, flexibility in molecular design, and lower production costs compared to those of inorganic compounds.^1,2^ These features have made them useful in photonic systems like all-optical switches, light amplifiers, organic light-emitting diodes (OLEDs), lasers, and many more.^3−9^

Among various organic materials, the ones deserving special attention are certainly acedan derivatives. The ADN core stands out as a versatile framework that is easily adaptable to incorporate diverse functional groups. Consequently, this enables the precise tunability of their spectroscopic properties. ADNs, classified as nonsymmetrical donor–acceptor (D–A) compounds, demonstrate exceptional characteristics that make them highly attractive for diverse applications. Their attributes, such as two-photon absorption, meet rigorous criteria for bioimaging applications.^10−13^ Furthermore, ADNs have received considerable attention as superb materials for designing fluorescent probes used to label biomolecules and organelles.^10,13−15^ Some of these derivatives show excellent solubility, outstanding photostability, high quantum yield, fluorescence, photochromism, and electrochromism.^16^ In this context, our research aims to show the distinctive properties of synthesized ADN derivatives (specifically, ADN1, ADN2, and ADN3) and propose new applications beyond those discussed in the current literature.

First, we detail the synthesis process of ADN compounds. Then, theoretical studies using TD-DFT techniques are demonstrated, concerning the conformational analysis, excited-state properties, and charge-transfer diagnostic. These insights provide a foundation for further empirical investigations. To highlight the versatility of ADNs, we performed NLO experiments involving THG and OKE. The results confirm all dyes’ exceptional third-order NLO responses in host–guest arrangements. Specifically, ADN1, ADN2, and ADN3 demonstrate 2.5-, 2.0-, and an impressive 12-fold increase in third-order NLO susceptibility (χ^(3)^) compared to the reference material (silica). OKE results showcase significant *trans–cis* isomerization occurring in ADN2 and ADN3, leading to temporary optical photoinduced birefringence (PIB), an essential feature for applications concerning reversible data storage and holographic recording.^17−19^

Finally, we demonstrate that a mixture of ADN derivatives can provide various colors of fluorescence. In more detail, by layering different chromophores in host–guest polymeric thin films, it is possible to shift the emission spectra from 440 nm (deep blue) to 520 nm (yellow-green). This ability to manipulate emission wavelengths is crucial for creating adjustable light sources, cutting-edge displays, and advanced microscopy techniques.^20−28^ Moreover, the precise tuning of the emitted colors, which is feasible/achievable for examined ADNs, grants numerous prospects for applications such as sensors and biological imaging, where multicolor fluorescence is highly desirable.^29−32^ To gain insight into the interactions occurring between the individual dyes, we analyzed fluorescence lifetime decays and explored the phenomenon of energy transfer concerning two- and three-chromophore arrangements.

The significance and novelty of our research aim to identify new properties of ADNs through carefully designed studies that integrate the synthesis, theoretical computations, and experiments. This holistic characterization offers fresh insights into ADNs, introducing functionalities not discussed in the literature.

## 2 Experimental Section

### 2.1 Thin-Film Preparation

#### 2.1.1 ADN Dyes: Synthesis

The chromophores investigated in this paper consist of 1-(6-dimethylaminonaphthalen-2-yl) ethanone (abbreviated here as ADN1), (E)-4-(6-(dimetyloamino) naftalen-2-yl)but-3-en-2-one (ADN2), and (E)-3-(6-(dimetyloamino)naftalen-2-yl)-1-fenylprop-2-en-1-one (ADN3). The slightly modified synthesis routes are demonstrated in the S1–S5 SI files^11,16^ together with ^1^H NMR, ^13^C NMR, FT-IR (ATR), and HR ESI-MS spectra for ADN1 (Section S5, Figures S1–S4), ADN2 (Figures S5–S8), and ADN3 (Figures S9–S12), respectively.

#### 2.1.2 Solution Preparation

First, a solution of poly(methyl methacrylate) (PMMA, purchased from Sigma-Aldrich) polymer was prepared by dissolving the powder in dichloromethane at a concentration of 5%.

For the THG experiment, dyes were added to each polymer solution at a content of 0.5% (weight by weight in dry mass). For the OKE experiment, the ADNs were doped into the PMMA matrix, maintaining a concentration of 0.5% (w/w). The difference in concentration between OKE- and THG-dedicated samples is discussed in detail in the next section.

#### 2.1.3 Sample Preparation

In preparation for the THG experiment, the described solutions were evenly deposited on the glass substrate to form thin films using the spin-coating (SC) method. The spinning parameters were set at a speed of 1000 rpm for 45 s. The resulting film thicknesses were measured as follows: 2.2 μm for ADN1, 2.1 μm for ADN2, and 1.9 μm for ADN3 (measured using a Dektak 6M).

Samples for OKE require thicker films to ensure more active species in the area of interest and space for molecular reorientation. Therefore, samples were prepared by the drop-casting method. A 0.5% (dye to polymer, w/w in dry mass) solution was poured onto a glass plate placed in a solvent atmosphere in a Petri dish, protected from sunlight. Samples were left untouched until the solvent fully evaporated. Lower concentrations prevent unwanted crystallization, which can occur during prolonged solvent evaporation, as well as ensure enough space within the sample for dynamic molecular reorientation of chromophores. Prepared samples had thicknesses of 27.8 and 26.2 μm for ADN2 and ADN3 respectively (measured using a profilometer, Veeco Dektak 3).

For the experiment focusing on polychromatic fluorescence and multicolor tuning of samples, a specific approach was employed. Initially, solutions were prepared by incorporating individual chromophores into a PMMA matrix (0.5% dye/polymer, w/w in dry mass). Then, to create the multiple color-emitting samples, a layer-by-layer SC method was utilized. This involved depositing the first layer (1000 rpm, 45 s) onto the glass substrate and allowing it to dry for 24 h before applying the next one. This process resulted in a sandwich-like system, where multiple (two/three) layers containing different dyes were stacked. In this paper, for example, a sample marked as ADN1-ADN2 indicates that the bottom layer is doped with ADN1 while the top layer contains ADN2.

### 2.2 Computational Details

All of the DFT and TD-DFT calculations were performed with the latest version of Gaussian 16 software^33^ using a 6-311G+(2d,p) atomic basis set.^34,35^ During the optimization procedure, the default self-consistent field convergence criterion was improved to 10^–10^ au while the optimization threshold was enhanced to 10^–5^ au on average residual forces. In all mentioned calculations, a so-called *ultrafine* pruned (99 radial shells and 590 angular points per shell) integration grid was employed. Based on the high predictive power presented by the MN15 functional^36^ for excited-state properties of the wide range of organic dyes,^37^ we selected this (TD)-DFT approach. First, the ground-state geometrical parameters were identified and confirmed by vibrational analysis, which proved that found structures correspond to true minima on ground-state potential energy surfaces (PES). Subsequently, the same procedure was followed for the excited-state characterization. The transition energies between the two related states have been determined at the TD-DFT and ADC2 (emission) levels of theory. To account for the conditions of experimental measurements (PMMA matrix), all described TD-DFT calculations were performed using the polarizable-continuum model (PCM)^38,39^ in its linear-response (LR)^40,41^ (optimization and vibrational analysis) or corrected-linear-response (cLR)^42^ (vertical transitions) variants. In more detail, we used dibutylether solvent (ε ≈ 3.05) characterized by a dielectric constant close to that of PMMA (ε ≈ 3.0). Standard cLR-TD-DFT protocol proved to be inefficient in the reproduction of the key features of ADN emissive bands, probably due to the significant charge-transfer character of related excited states. Therefore, we followed the correction scheme proposed by the Jacquemin group^43^ involving a single-point correction from feasible post-Hatree-Fock methods (herein algebraic diagrammatic construction variant ADC2) and improving the description of solvent polarization by taking into account state-specific effects. The aforementioned ADC2 calculations were executed by applying the resolution of identity (RI) technique (using a triple-ζ auxiliary basis set) as implemented in the Turbomole 7.6 program.^44^ Lastly, density difference plots (DDPs) together with charge-transfer parameters were evaluated to gain additional insight into the nature of analyzed transitions. The above results were obtained using PCM-TD-MN15 adopting the methodology proposed by Le Bahers and co-workers.^45,46^ The mentioned DDPs were generated using 0.002 au contour values. In these graphs, the blue (red) areas indicate a density depletion (gain) upon photon absorption.

### 2.3 THG Studies

For the THG measurement setup, a picosecond pulsed Nd:YAG laser (EKSPLA, PL2250 series) with a 30 ps pulse duration and a 10 Hz repetition rate was exploited, emitting a 1064 nm wavelength. The laser beam was controlled by a polarizer, half-wave plate (for polarization control), filter, and focusing lens before it interacted with a sample on a rotating table. Filtering removed any residual second harmonic radiation. The sample could rotate from +60° to −60° relative to the incident beam and the normal direction. Data was collected by varying angles in 0.5° step intervals. Gray and interference filters ensured precise wavelength and intensity control. A photodetector and computer program analyzed the resulting beam, with stability monitored by an oscilloscope. Laser energy (100 μJ) was measured using a Coherent-Field Max II power meter. Please note that THG is independent of the polarization configuration. For simplicity and clarity, we present the results only for the s-polarization configuration.

The determination of χ^(3)^ was performed in a step-by-step procedure, starting with the measurement of the bare silica substrate as a reference material with its well-known χ^(3)^ value. After that, measurements were taken for the glass substrate (Maker fringes pattern in Figure S13) to ensure that the glass did not contribute significantly to the THG signal. Finally, the sample composed of the same glass substrate, onto which a polymer layer containing the chromophore (dye) was deposited, was measured. To isolate the contribution of the polymer film from the total THG signal, the signal from the glass substrate was subtracted. This correction ensured that the final value for χ^(3)^ represented the NLO of the thin polymer film containing the ADN chromophores. This approach follows standard practices for measuring third-order nonlinear susceptibilities of thin films deposited on substrates, as outlined in the work by Kajzar et al.^47^

Determining the third-order nonlinear susceptibility (χ^(3)^) in THG experiments is crucial, as it quantifies how materials respond to light fields. For the calculation of the χ^(3)^ value, the comparative^48−50^ model was employed, which is valid for the thin polymeric films. When estimating χ^(3)^, it is important to consider whether the absorption at the 355 nm wavelength is significant. In the case of ADNs, this parameter cannot be overlooked and should be considered in calculations as well as the optical transparency to avoid damage (Section S4 and Figure S14). Therefore, the analyses were executed by utilizing the following formula^51^
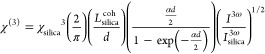
where χ_silica_^3^ = 2.0 × 10^–22^ (m^2^/V^2^).^52,53^*L*_silica_^coh^ = 6.7 μm is the coherence length of silica, calculated with the equation
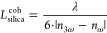


Here, *n*_3ω_ and *n*_*ω*_ represent the refractive indices of the material at the tripled frequency (1.4761 at 355 nm) and fundamental (1.4496 at 1064 nm) wavelengths, respectively. In the equation, *d* is the sample thickness (μm) and α is the absorption coefficient at 355 nm (cm^–1^). *I*^3ω^ and *I*_silica_^3ω^ are maximum THG intensities of the sample and silica, respectively, obtained from the measurement (Arb. Un.).

### 2.4 PIB

The PIB was measured using the experimental setup for the OKE.^54^ This approach represents a typical pump–probe experiment, enabling the observation of temporary induced anisotropy in an initially isotropic medium. That includes both dynamic and static changes in the refractive index as well as signal growth and decay. Through this method, it is possible to estimate the PIB (*Δn*), nonlinear refractive index (*n*_*2*_), and third-order nonlinear optical susceptibility (χ^(3)^). The continuous wave DPSS laser (λ = 405 nm) was used as the pump beam, as its wavelength lies within the absorption spectrum of all of the investigated ADN derivatives. For monitoring the PIB, a He–Ne laser operating at 632.8 nm was selected as the probing beam. The polymeric thin film was placed between two crossed polarizers, so the transmittance of the probing beam depends on the induced birefringence within the system. To eliminate the residual pumping beam, an interference band-pass filter transmitting only the probing beam (632.8 nm) was employed. The calculation of the optical birefringence value (*Δn*) utilized the following relation
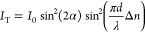
where *I*_T_ is the transmitted light intensity recorded by the photodiode, *I*_0_ is the initial intensity of the probing beam, α represents the angle between the polarization of pump and probe beams, λ is the probing beam wavelength, and *d* is the sample’s thickness. A photoinduced change in the refractive index is directly proportional to the pumping light intensity *I*_pump_ and can be defined as follows
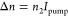
where *n*_2_ denotes the nonlinear refractive index, which furthermore can be used for the estimation of the third-order susceptibility using the following relation:
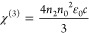


Here, *n*_0_ represents the refractive index of the sample in its isotropic state, *c* denotes the speed of light, and *ε*_0_ is the vacuum electric permittivity.

### 2.5 Multicolor Emission, Energy Transfer, and Fluorescence Lifetime Studies

The absorbance spectra of single-, double-, and triple-dye sample sets embedded in the PMMA matrix were recorded at ambient temperature by using a UV-1800 Shimadzu spectrophotometer. The fluorescence studies were performed by employing a Fluoromax-4 Horiba spectrofluorometer with an excitation wavelength (λ_exc_) of 390 nm. Both absorbance and emission spectra were obtained by ensuring spectral resolutions of 0.5 and 1.0 nm, respectively.

The 1931 CIE XYZ model was selected for color analysis because of its common use in color science and its reasonable approximation of human color perception.^55−57^ In this model, tristimulus values (*X*, *Y*, and *Z*) represent the amounts of red, green, and blue (RGB) needed to create a color. Chromaticity coordinates (*x*, *y*, *z*) can be calculated from tristimulus values using specific formulas:
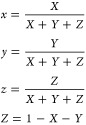


Luminescence lifetime measurements were performed with spectrofluorometer Edinburgh Instruments FLS980 equipped with 280 and 360 nm laser diodes. Measurements for single and multiple dye-doped thin films were carried out. Decay-time measurements were performed using excitation wavelengths equal to 280 and 360 nm and different observed emission wavelengths depending on the tested compound in the mixture (for ADN1 it was set to 430 nm, for ADN2 we chose 500 nm, and finally for ADN3 it was 520 nm).

## 3 Results and Discussion

### 3.1 Quantum Chemical Calculations

Computational characterizations of ADNs started with the investigation of the most stable rotamers (Table 1). In the case of ADN2 and ADN3, two template structures were proposed by considering the rotation around the single C–C bond.

**Table 1:**
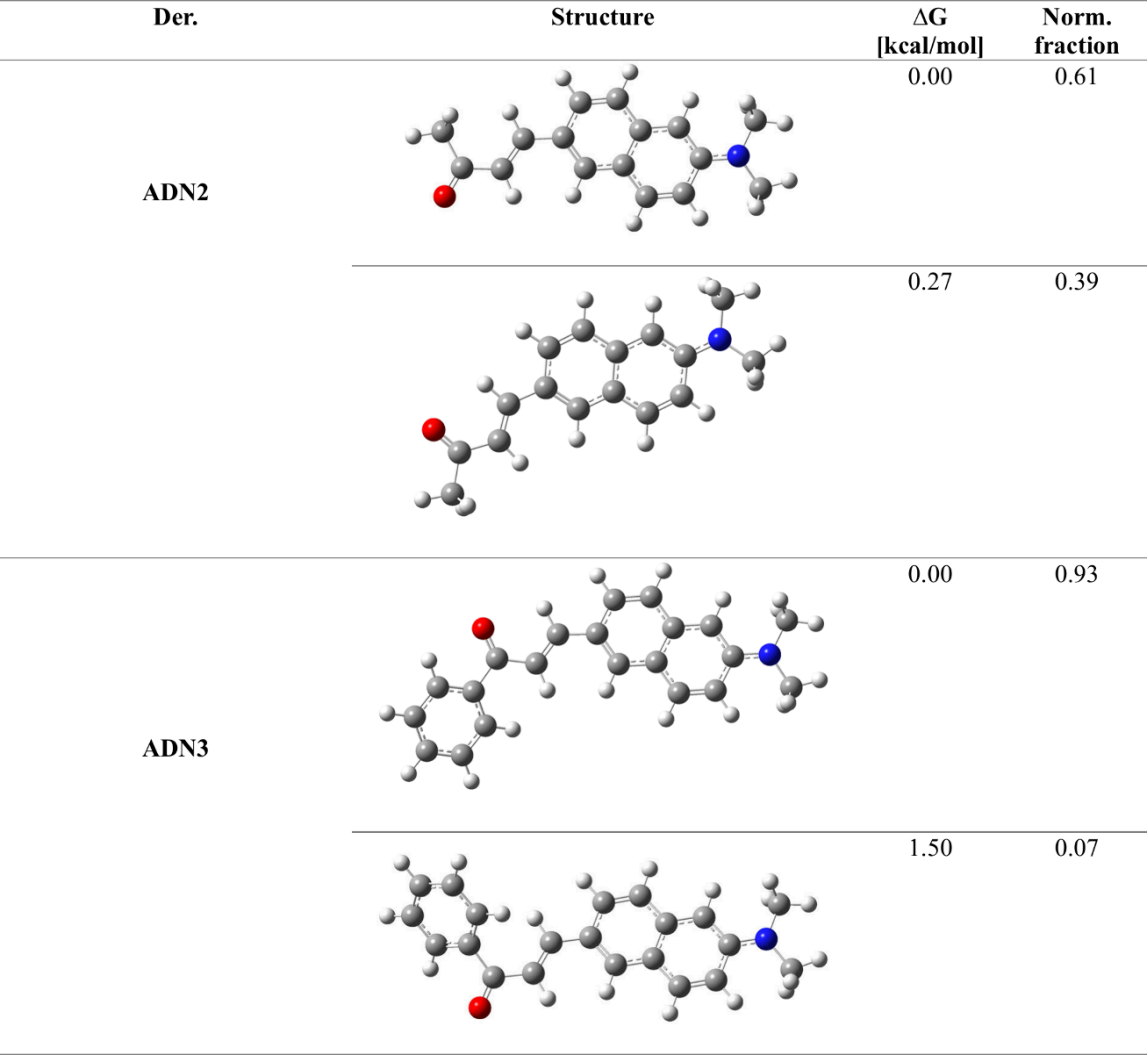
Conformational Analysis^a^

a Δ*G* describes relative Gibbs free energies computed at the MN15/6-311G+(2d,p) theory level. Rotamer fractions were calculated by employing the Boltzmann distribution formula at 298 K.

The difference between relative free Gibbs energies of ADN2 conformers was relatively small (0.27 kcal/mol), thus on the grounds of the Boltzmann distribution (and assuming room temperature), both conformers are present within the medium, with the dominating rotamer constituting 61% of the ratio. Therefore, both ADN2 rotamers will be included in the discussion below. In turn, for ADN3, the difference between relative free Gibbs energies was quite significant (1.50 kcal/mol), which is a consequence of spherical hindrance generated by the phenyl ring in the second ADN3 rotamer. Subsequently, a single predominant conformer (constituting a 0.93 fractions of the mixture) will be analyzed.

To validate the accuracy of the selected computational protocol, the TD-DFT predictions were compared with the experimental measurements (Figure S3, absorbance and fluorescence of prepared thin films). As illustrated in Table 2, the chosen approaches adequately predict the position of the absorption and emission bands. In the case of absorption bands, the prediction errors do not exceed 10 nm, perfectly reproducing the increasing red-shift trend arising from intramolecular charge transfer following the structural modification introduced into the series, i.e., extending the linker by a double C–C bond (ADN1 → ADN2) and/or by adding a phenyl ring (ADN1 → ADN3). Described increasing red-shift trends are even more pronounced in the emission bands, which are also (to some extent) reproduced by simulations. Nevertheless, the predicted position of the ADN1 emission band is a bit underestimated, which is reflected by excessive values of red-shift estimations (with respect to trends observed in the measured spectra). In the case of ADN2, it can be seen that both stable conformers bear similar photophysical characteristics, i.e., transition maxima position and related oscillator strengths; however, the emission band related to the dominant conformer is slightly (20 nm) more red-shifted.

**Table 2:** Comparison between Measured Absorption and Emission Band Maxima and Related Computed Transition Energies with Respective Oscillator Strengths (*f*)^a^

			Theory			
	Exp		MN15-cLR-TD-DFT		MN15-(cLR+LR)-TD-DFT+ADC2	
Lp	λ_max_^abs^	λ_max_^fl^	Λ_theo_^abs^	*f*	Λ_theo_^fl^	*f*
ADN1	352	440	346	0.44	421	0.49
ADN2	387 (+35)	486 (+46)	385 (+39)^b^	0.78^b^	496 (+75)^b^	1.01^b^
			389 (+43)	0.81	476 (+55)	0.96
ADN3	415 (+63)	520 (+80)	412 (+66)	0.93	517 (+95)	1.12

a Red shifts with respect to ADN1 values are given in parentheses.

b More stable conformers.

The TD-DFT analysis revealed that the main absorption bands correspond to the S_0_ → S_1_ transitions, while weak (oscillator strengths ca. 0.1) S_0_ → S_3_ transitions around 315–330 nm may play a role in the broadening of absorption spectra tails. Lowest-lying π–π* transitions of ADN2 and ADN3 are characterized by fairly large oscillator strengths (*f*) exceeding 0.8, while a much lower *f* was noted for ADN1 (0.42). For all dyes, the highest contributions to main absorption transitions can be ascribed to one-electron HOMO → LUMO excitation; however, non-negligible fractions from HOMO → LUMO + 1 transitions are also present.

To gain additional insight into the nature of the lowest-lying π → π* transitions, ICT (intramolecular charge transfer) characterization was performed. As can be seen from electron density difference (EDD) plots (Table 3), the NMe_2_ units act as an electron donor, while C(O)CH_3_/C(O)Phe groups serve as an electron acceptor for all examined derivatives, which is consistent with our preliminary assumptions concerning the D−π–A nature of synthesized compounds. Surprisingly in the case of ADN3, the attachment of an additional phenyl ring to the acceptor side does not greatly affect the electron density flow. As shown on the EDD plot (Table 3), only minor red orbs appear on the aforementioned phenyl ring, which indicates that it serves as the weak secondary acceptor. All examined transitions display a strong CT nature, which is reflected by large dipole moment difference values (μ_ES_ – μ_GS_), reaching 14 D for ADN3. Notably, all examined CT metrics (μ_ES_ – μ_GS_, *d*_CT_, and *q*_CT_) are systematically increasing following the π-conjugation extension pattern within the series (ADN1 → ADN2 → ADN3); however, the extent of the rise depends on the property. The metric that is the most sensitive to elongating and extending the π-conjugation path is change within dipole moments, as the μ_ES_ – μ_GS_ value is almost doubled between the original and most extended molecules within the series (ca. 7 and 14 D for ADN1 and ADN3, respectively). Naturally, the charge-transfer distance is also substantially increased by π-conjugation extension; that is, the *d*_CT_ value goes from 2.74 Å in ADN1 to 4.55 Å in ADN3, while *q*_CT_ values are only slightly affected (*q*_CT_ increased by merely 0.1 e between ADN1 and ADN3). The CT nature of the S_0_ → S_1_ transitions related to both ADN2 rotamers bears a strong resemblance; however, the dominant conformer is characterized by a slightly higher dipole moment change (ca. 0.3 D).

**Table 3:**
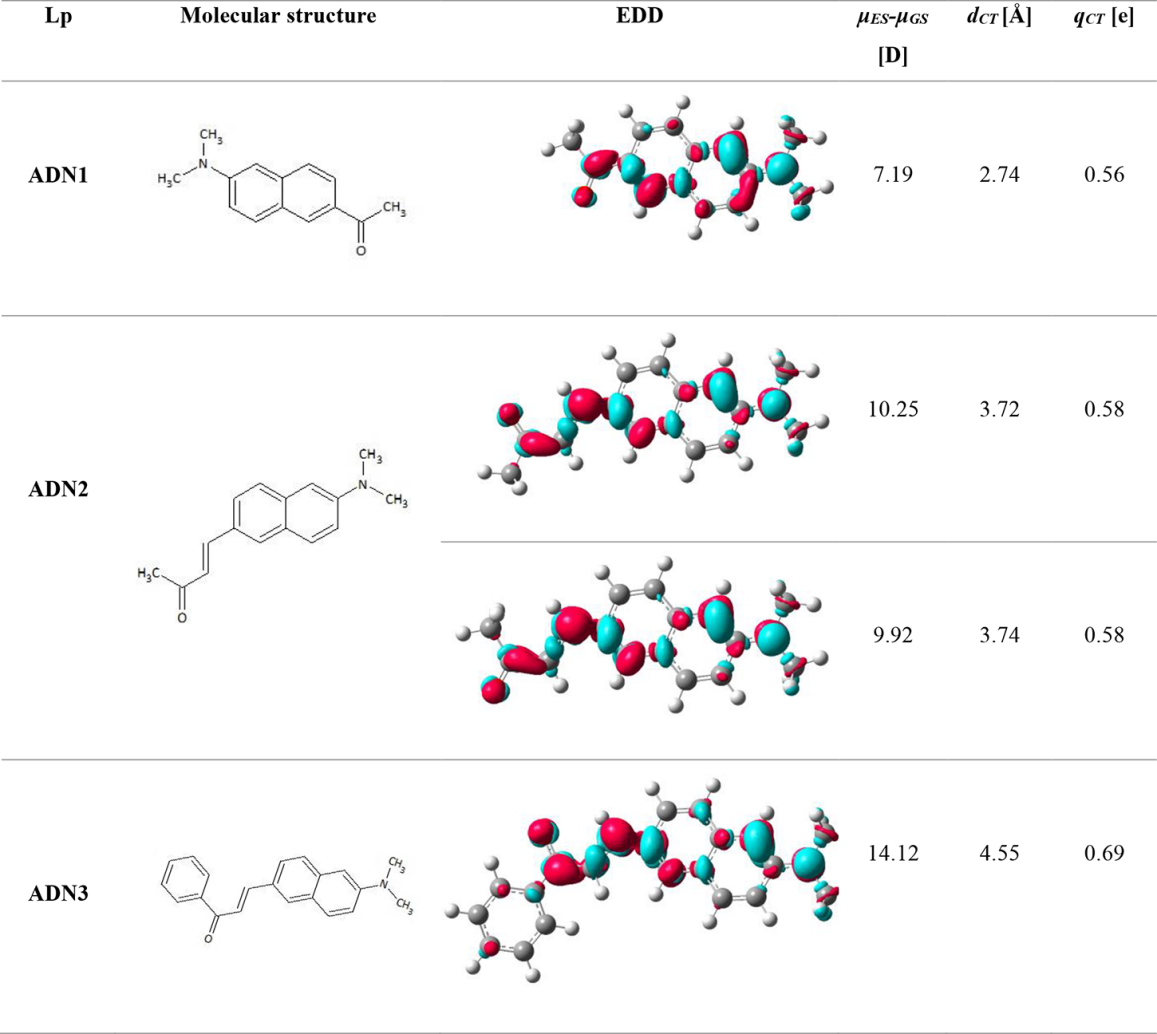
Electron Density Difference (EDD) Plots and Related Electron Transition Parameters of ADNs^a^

a *d*_CT_ stands for charge-transfer distance, *q*_CT_ is the total transferred charge, and μ_ES_ – μ_GS_ represents the difference in the dipole moment magnitude between the ground and Franck-Condon region of the excited state. In the EDD plots, the blue (red) color marks electron density depletion upon photon absorption. The contour value was set to 0.002 Arb.Un.

### 3.2 THG Studies

**Figure 1:**
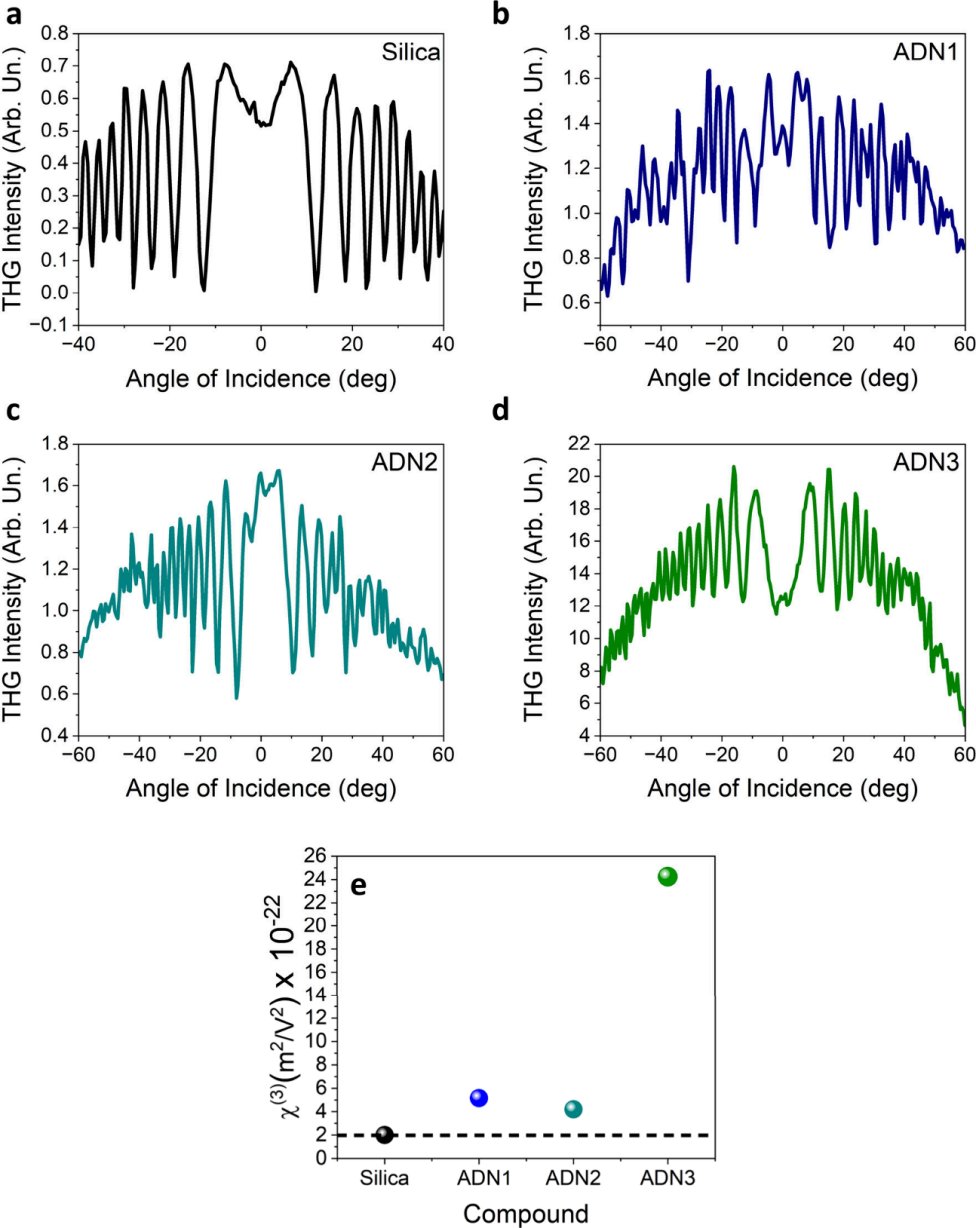
THG signal in the shape of Maker fringes for the reference material (silica) (a) and investigated NLO chromophores, namely, ADN1 (b), ADN2 (c), and ADN3 (d). Comparison of all of the obtained χ^(3)^ values (e).

ADN derivatives feature the naphthalene core with two substituents (acetyl and dimethylamino groups in the 2- and 6-positions, respectively) possessing contrasting electronic characteristics, strategically attached in opposing orientations. The photophysical properties of this common structural motif can be clarified through ICT processes. As shown in the Computational Details section (Discussion and Table 3), the electron density perturbation occurs via a π-electron linkage connecting the electron-donating to the electron-withdrawing moieties. Consequently, ADN1 exemplifies a prototypical case of organic fluorophores incorporating a donor–bridge–acceptor (D−π–A) system, making it very appealing for NLO studies. The system indeed demonstrates good signal modulation, seen as the Maker fringes for all three discussed compounds (Figure 1). The THG signal of the glass is demonstrated in S3 and Figure S13. First, there is highlighted the interference pattern for a benchmark, silica (1a), then ADN1 (1b), ADN2 (1c), and ADN3 (1d).

ADN2, compared to ADN1, exhibits stronger ICT character due to the extension of the π-system (C=C bond). The π-orbital of the acetyl group can accept electron density from the conjugated system, making it an acceptor region, as was presented on the EDD plots (Table 3). Further extension of the π-system through the replacement of the methyl group with a phenyl ring in the acceptor moiety of ADN2 gives rise to ADN3.^11^ As expected, the increased conjugation length and the benzene ring in these systems facilitate electron cloud delocalization even further, leading to a stronger THG response in ADN3. In this regard, the χ^(3)^ value is calculated (refer to the methodology outlined in the Experimental Section) and is approximately 12 times better than that of the silica reference (24.24 × 10^–22^ (m^2^/V^2^)). Nevertheless, the signals observed for ADN1 and ADN2 also demonstrate favorable outcomes, exceeding 2.5- and 2.0-fold enhancement (5.15 × 10^–22^ and 4.17 × 10^–22^ (m^2^/V^2^), with respect to silica (2.00 × 10^–22^ (m^2^/V^2^) (Figure 1e and Table 4).

**Table 4:** Third-Order Nonlinear Susceptibilities and Thicknesses of All of the Investigated Thin Films

Compounds	*d* (μm)	χ^(3)^ × 10^–22^ (m^2^/V^2^)	ref
**Silica**	1000	2.00	(50)
**ADN1/PMMA**	2.2	5.15	This work
**ADN2/PMMA**	2.1	4.17	This work
**ADN3/PMMA**	1.9	24.24	This work
**PTVTald/PMMA**	9.15	4.79	(58)
**PerRED/PMMA**	1.32	4.069	(59)
**Aurone-based polymer P2**	0.3	4.2	(60)
**Aurone-based polymer P1**	0.1	226.1	(60)
**Benzonitryle sample C/PMMA 5%**	0.4	12.26	(61)
**Benzonitryle sample C/PMMA 10%**	0.2	20.84	(61)

Recently, Szukalski et al.^58^ reported third-order NLO susceptibility values for (E)-5-(2-(5-phenylthiophen-2-yl)vinyl)thiophene-2-carbaldehyde (namely PTVTald) embedded in PMMA using the Maker fringe technique. Compared to ADN/PMMA samples, PTVTald/PMMA contains a thiophene-based core, which is characterized by high polarizability and conjugation and may support strong charge transfer. However, despite this conjugation, this sample exhibits a lower response than ADN3/PMMA. This suggests that ADN3/PMMA has a better donor–acceptor configuration compared to that of PTVTald/PMMA. This effect is stronger in the electron-donating dimethyloamino group and extended π-conjugation in ADN/PMMA samples, compared to the thiophene-based core and aldehyde group, which in this case are less effective in PTVTald/PMMA. Moreover, Szukalska et al.^59^ reported THG studies on perylene derivatives. PerRED (perylenetetracarboxylic dianhydride) embedded in PMMA, which exhibits the strongest NLO performance in this work, is characterized by a conjugated aromatic system with a perylene core and an electron-withdrawing anhydride group. The lack of an electron-donating group in PerRED/PMMA limits its effectiveness in third-order NLO properties, as we observe in the ADN3/PMMA case. Moreover, PerRED/PMMA has a more planar structure, compared to ADN/PMMA samples, and less extended π-conjugation, which minimizes its THG performance. Furthermore, Waszkowska et al.^60^ reported NLO insights into aurone-based methacrylic polymers. The third-order NLO susceptibility values reported for the P2 aurone-based polymer are close to those of the ADN2/PMMA sample. This polymer exhibits weaker donor–acceptor interactions due to a smaller dipole moment (4.71 D) compared to that of polymer P1 (8.71 D). We observe that further modifications in the molecular structure can enhance the NLO performance; polymer P1 contains a dimethylamino group (as in the ADN/PMMA case), which acts as a donor, and benzofuran, which acts as an acceptor. In this configuration, a strong donor–acceptor interaction enhances charge transfer and polarizability in the system, which leads to a strong NLO response. Besides, Mydlova et al.^61^ reported THG studies on (Z)-4-(2-(4-(9H-carbazol-9-yl)phenyl)-1-cyanovinyl)benzonitryle (namely C) in different concentrations: 5 and 10% of the material related to the polymer solution. This sample contains a carbazole group, characterized as an electron donor with a delocalized π-system, a benzonitrile group, which acts as an electron acceptor, and a phenyl-1-cyanovinyl linker, which features the push–pull design. We observe that the ADN3/PMMA 2% sample has a stronger NLO response than both C/PMMA 5 and 10% concentrations. The dimethylamino group in ADN3/PMMA is a stronger donor than the carbazole group in C/PMMA; moreover, the charge transfer is more effective in ADN3/PMMA than in phenyl-1-cyanovinyl C/PMMA. However, what caught our attention is the fact that increased molecular density at 10% likely boosted intermolecular interactions in C/PMMA, resulting in stronger THG properties. These comparative analyses underline the role of molecular design, especially the electron donor–acceptor interplay (dimethylamino group vs thiophene-based core/perylene core/benzonitrile group), extended π-conjugation length, dipole moments enhanced by specific moieties (i.e., benzofuran), and dye concentration in the polymer matrix in achieving the strongest THG properties in NLO materials.

### 3.3 PIB

Only two of the three investigated compounds have a double bond in their structures, which allows dynamic *trans–cis* isomerization. Moreover, when subjected to linearly polarized laser light, molecules can additionally reorient perpendicularly to the electric field vector and undergo conformational changes.^62−64^ Such phenomena can induce molecular alignment, resulting in temporary so-called static optical birefringence.

**Figure 2:**
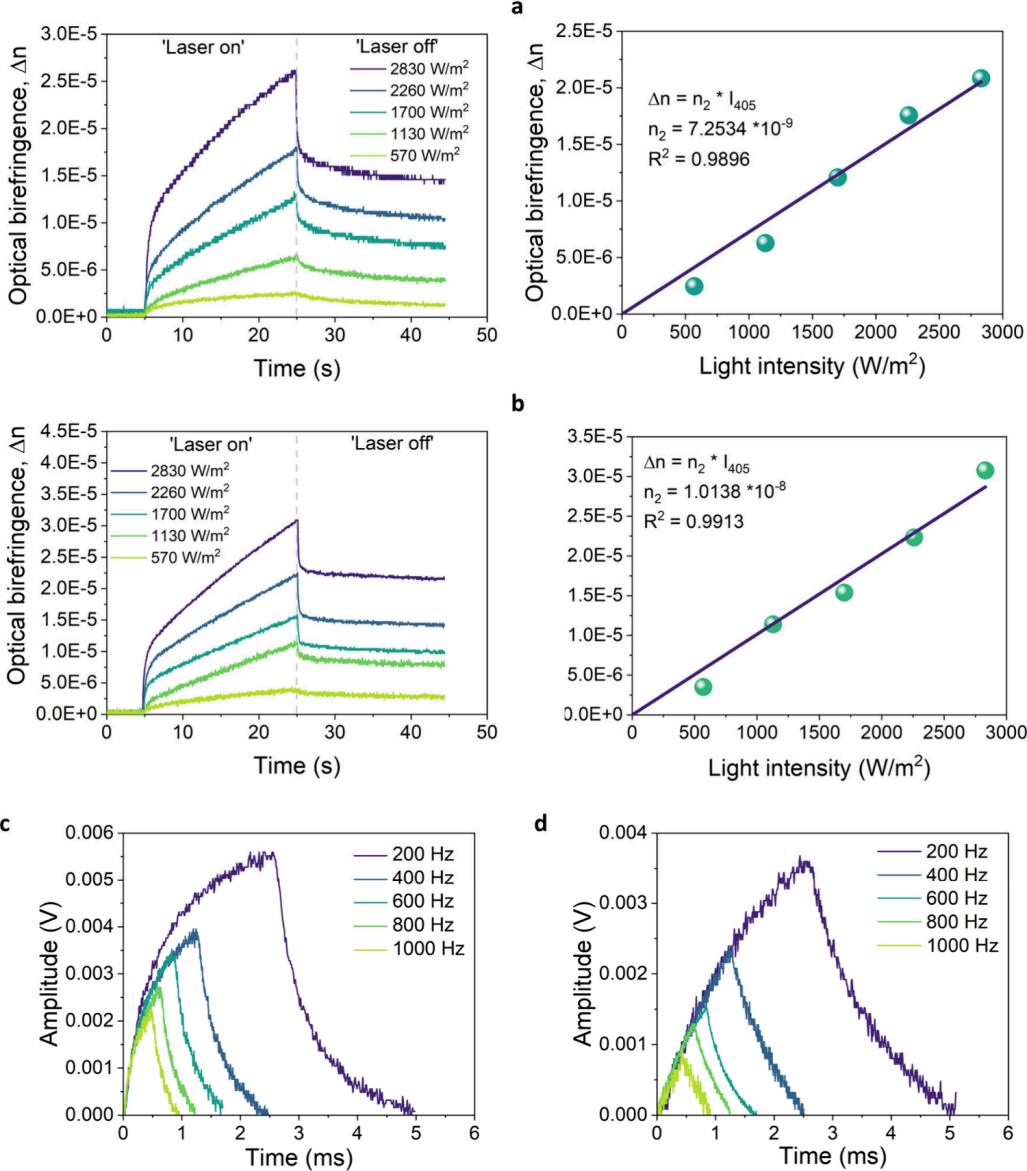
Kinetics of PIB with the corresponding linear relation of Δ*n* as a function of pumping laser beam intensity for the system with ADN2 (a) and ADN3 (b). Dynamic change in the refractive index induced by different chopper frequencies for ADN2 (c) and ADN3 (d).

The OKE experiment was segmented into two parts to monitor the behavior of the molecules across different timeframes. Initially, the static PIB was estimated, wherein the sample was continuously exposed to the incident beam for a selected time duration. Afterward, the laser light was blocked to monitor the thermal relaxation of the system. Both molecules (ADN2 and ADN3) express similar responses where growth and decay of the signal can be fitted with a biexponential, indicating two internal processes occurring in the sample. The first process arises significantly faster than the other, leading to gradually increasing PIB even after 25 s of illumination. Similar behavior can be seen for signal decay. At the end of the measurement, residual PIB remains in the sample, which is quite common behavior for organic systems and was already recorded for azobenzene and spiropyran derivatives.^65,66^ The obtained results are very similar, although a slightly higher signal in the case of static change was recorded for sample ADN3; consequently, the calculated NLO coefficients are also higher (Table 5). In the case of nonlinear susceptibility, the χ^(3)^ calculated values are 5.78 × 10^–11^ (m^2^/V^2^) and 8.08 × 10^–11^ (m^2^/V^2^) for ADN2 and AND3, respectively, which falls in a typical range for organic chromophores. The value of the nonlinear refractive index was estimated by a linear fit of PIB as a function of the pumping beam power leading to obtaining values equal to 7.25 × 10^–9^ (m^2^/W) for ADN2 and 1.01 × 10^–8^ (m^2^/W) for ADN3 (Figure 2a,b).

**Table 5:** Summary of the OKE Results^a^

System	Δ*n*_static_	*n*_2_ (m^2^/W)	χ^(3)^ (m^2^/V^2^)	*Τ*_gr._^st.^ (s)	*Τ*_dec._^st.^ (s)	τ_gr_^dyn.^(μs)	τ_dec._^dyn.^(μs)
ADN2 in PMMA	2.60 × 10^–5^	7.25 × 10^–9^	5.78 × 10^–11^	0.47/26.27	0.66/4.15	386	305
ADN3 in PMMA	3.07 × 10^–5^	1.01 × 10^–8^	8.08 × 10^–11^	0.32/40.16	0.47/42.42	752	439

a st. stands for “static”; gr., “growth”; dec., “decay”; and dyn., “dynamic”.

The second part of the OKE experiment focused on monitoring the fast intramolecular changes of the sample upon illumination with a pump beam operating at a selected frequency. In such a short time regime it was possible to observe the kinetics of *trans–cis–trans* photoisomerization. The experiment was conducted with light modulation ranging from 200 to 1000 Hz. Both systems exhibit a fast and profound change in the signal amplitude even at 1000 Hz of choppering frequency (Figure 2c, and d). The visible decrease between consecutive measurements can be accounted for by the overall lower energy applied to the system when the choppering frequency is increased. Analysis of the recorded kinetics suggests that in ADN2 *trans–cis–trans* photoisomerization occurs faster than in the case of ADN3. This can be explained by the more extensive structure of ADN3 which contains an additional phenyl ring, which generates significant spherical hindrance, thus making conformational changes slower. Unlike the outcomes of SHG or THG experiments, the results of PIB are not heavily dependent on the extent of ICT. Instead, the observed trends in PIB may be better explained by the Kramers-Krönig relation. In the OKE experiment, the selection of the pump beam wavelength significantly influences the obtained results, as the number of absorbed photons contributes to the induced changes in refractive index. Consequently, the tabulated (Table 5) results are valid only for the specified pump wavelength of λ_pump_ = 405 nm.

### 3.4 Multicolor Fluorescence and Energy-Transfer Studies

**Figure 3:**
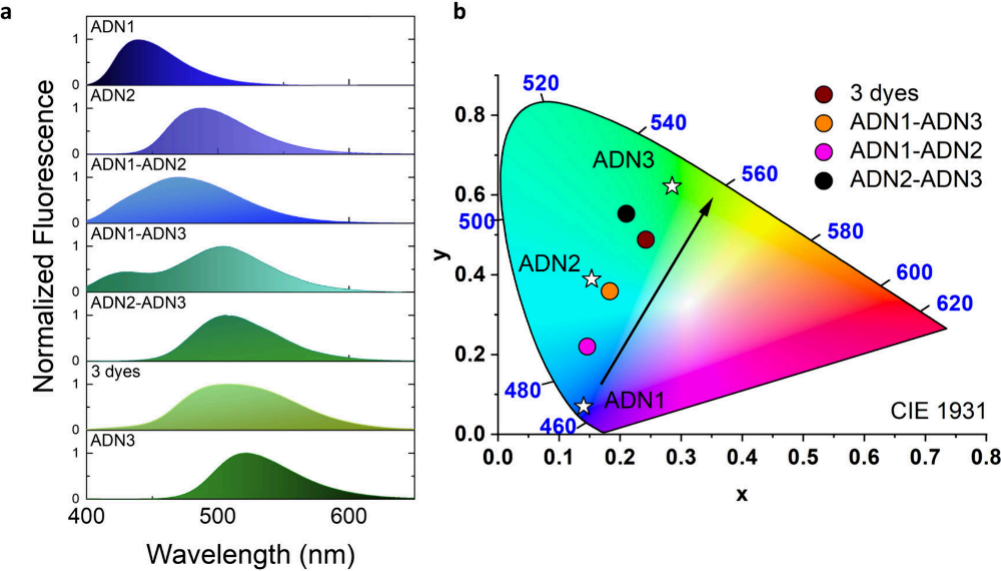
Collective fluorescence spectra of one-, two-, and three-dye thin-film systems (a). CIE XYZ 1931 model demonstrating color tunability (b).

Figure 3a illustrates multicolor fluorescence tuning. For the comparative analysis, we examined the spectra of individual dyes from the ADN family, as well as those from the mixed systems.

In the case of the ADN1-ADN2 pair, the fluorescence spectrum shows broadening, ranging from 400 to 600 nm with a single peak around λ_max_ = 470 nm. The CIE XYZ diagram (Figure 3b) reveals that this point lies between these two single dyes and corresponds to a light blue color. For the ADN1-ADN3 system, two peaks emerge: one with the highest intensity at a wavelength of λ_max_ = 505 nm (attributed to ADN3) and another at λ_max_ = 430 nm (originating from ADN1). The fluorescence range remains consistent with the previous case; however, the position of the point on the chromaticity triangle has shifted toward the blue-green region. Interestingly, the trichromatic coordinates in this scenario closely resemble that obtained for the single ADN2 dye (Figure 3b). The CIE XYZ point for the system containing 3 dyes has shifted further toward the green region, displaying a mint hue. Additionally, a notable red shift of the fluorescence spectrum maximum (λ_max_ = 511 nm, emission range: 425–630 nm) is observed. The ADN2-ADN3 pair, which exhibits the emission closest to that of individual ADN3, is the one that is shifted most toward green emission. This is justified by the absence of a spectral contribution of a dye that would predominantly provide blue light (ADN1). This observation may be assigned to a possible energy transfer process, occurring in such circumstances. In this case, the emission maximum occurs at 505 nm, with a significantly wider range than the three dyes case, encompassing a substantial portion of the green light emission range (435–600 nm).

Due to the chosen methodology (Experimental Section) to obtain the two- and three-dye systems within the polymer matrix, the potential for the proximity of neighboring chromophores must be considered. The sequential deposition of each layer allows for such a process to occur, thereby enhancing the likelihood of Förster resonance energy transfer (FRET) through dipole–dipole interactions. Also, the SC method used during the layer deposition process ensures uniform and controlled layer thickness, resulting in optimizing the distances between the chromophores. Moreover, upon analyzing the overlap between the absorbance and fluorescence spectra, it can be deduced that the 2-dye system contains molecules exhibiting a donor–acceptor relationship. One of the crucial factors enabling FRET is the appropriate overlap between the emission spectrum of the donor dye and the absorption spectrum of the acceptor dye (SI file, Section S5, Figure S15). It can be concluded that ADN1 acts as a donor when paired with either ADN2 or ADN3. ADN2 may also serve as a donor for ADN3, while the latter chromophore most likely acts as an acceptor within the systems. To confirm these assumptions, an experiment was conducted to measure the fluorescence decay time for the donor both individually and within all of the blends. The results were analyzed using a formula capable of describing the FRET efficiency (ϕ)
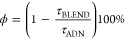
where *τ*_ADN_ is the fluorescence lifetime of ADN1 dye without the presence of any acceptor dyes and *τ*_BLEND_ is the fluorescence lifetime for ADN1 in two or tricolor blends.

The fluorescence lifetime decay curves are demonstrated in the ESI file, Section S6 and Figure S16. Table 6 displays the differences between the decay lifetime of ADN1 in the single-, pair-, or three-color systems.

**Table 6:** Fluorescence Lifetime and the FRET Efficiency of ADN1 in the Investigated Mixtures

Thin films	τ (ns)	Φ (%)
**ADN1/PMMA**	4.0	N/A
**ADN1-ADN2/PMMA**	2.8	30.5
**ADN1-ADN3/PMMA**	2.4	40.4
**three colors/PMMA**	1.9	52.9

In the case of the ADN1-ADN2 pair, an energy transfer of 30.5% can be observed, which is evident in the fluorescence spectrum of the related blend, as the peak intensity is shifted toward ADN2 (Figure 3a). In turn, calculated results indicate that for ADN1-ADN3 pair, this process occurs with a higher Φ, specifically 40.4%, which is noticed since the spectral band originating from ADN1 exhibits lower intensity compared to that attributed to ADN3. In the case of the three dye samples, the efficiency of the FRET process reaches a remarkable 52.9%, thereby explaining the attenuation of the spectral band originating from ADN1 in the spectrum (Figure 3a).

## 4 Conclusions

Our study demonstrates the multifunctionality and unique properties of ADN derivatives, namely, ADN1, ADN2, and ADN3 - through a comprehensive approach integrating synthesis, theoretical analyses, and experimental investigations. We have shown the remarkable performance of ADNs in NLO phenomena such as THG and OKE. The significant enhancement of the χ^(3)^ observed in ADN1, ADN2, and ADN3 compared to reference material emphasizes their potential for efficient frequency converters. In turn, the results of the OKE experiments establish the possibility of achieving ultrafast optical switching and modulation in ADN2 and ADN3, highlighting their potential for creating compact, integrated, and all-optical signal processing devices. Furthermore, our exploration into the polychromatic properties and tunability of the fluorescence color in ADN derivatives reveals promising applications in adjustable illumination sources, displays, and advanced microscopy techniques. The strategic layer-by-layer configuration of the chromophores enables the precise manipulation of emission hues, underlining the versatility of ADNs. Our findings not only contribute to expanding the understanding of ADNs but also highlight their novel functionalities that have not been discussed in the literature. Through experimental analyses and systematic characterization, we have identified new avenues for utilizing ADNs in diverse optoelectronic applications.

## Data Availability

The authors state that the data supporting the findings of this study are included within the paper and its Supporting Information (SI) files. Any raw data required in a different format can be obtained from the corresponding author upon reasonable request. Source data are also provided with this paper.

## Supporting Information Available

Additional experimental details, materials, and methods, in particular, (1) chemical synthesis and characterization of acedan dyes including ^13^C NMR, FT-IR, HR ESI-MS, and ^1^H NMR spectra; (2) THG response of the glass plate; (3) basic spectroscopic properties of thin films containing ADN1, ADN2, and ADN3 in the PMMA matrix; and (3) fluorescence lifetime decays of single ADN1 and ADN1 in double- and three-dye arrangements in PMMA (PDF)
