# Supplementary material for: From Theory to Practice: Advanced Nonlinear Optics and Multicolor, Tunable Fluorescence of Acedan Dyes
Source: J Phys Chem B. 2025 Feb 25;129(9):2514–25. doi: 10.1021/acs.jpcb.4c07533 (PMC11891890; doi:10.1021/acs.jpcb.4c07533)
Supplement: Supplementary file 1 — jp4c07533_si_001.pdf [file jp4c07533_si_001.pdf]

Electronic Supplementary Materials

From Theory to Practice: Advanced Nonlinear Optics and Multicolor, Tuneable Fluorescence of Acedan dyes

Alina Szukalska [1], Anna Grabarz [1,2], Bartłomiej Potaniec [3], Maria Zdończyk [3,4], Anna Popczyk [5], Karolina Waszkowska [6], Houda El Karout [6,7], Joanna Cybińska*[3,4], Bouchta Sahraoui [6], Jarosław Myśliwiec [1]*

[1] *Soft Matter Optics Group, Wrocław University of Science and Technology, Wyb. Wyspiańskiego 27, 50-370 Wrocław, Poland*
[2] *Department of Physical and Theoretical Chemistry, Faculty of Natural Sciences, Comenius University, Ilkovičova 6, 84215 Bratislava, Slovakia*
[3] *Łukasiewicz Research Network – PORT Polish Center for Technology Development, ul. Stabłowicka 147, 54-066 Wrocław, Poland*
[4]*University of Wrocław, Faculty of Chemistry, ul. F. Joliot-Curie 14, 50-383 Wrocław, Poland*
[5]*Humboldt Centre for Nano- and Biophotonics, Department of Chemistry, University of Cologne, Cologne, Germany*
[6] *Univ Angers, LPHIA, SFR MATRIX, F-49000 Angers, France*
[7] *Univ Angers, CNRS, MOLTECH-ANJOU, SFR MATRIX, F-49000 Angers, France*

[@] Corresponding Authors: alina.szukalska@pwr.edu.pl; joanna.cybinska@port.lukasiewicz.gov.pl

S1. General Information for Synthesis

All the chemical reagents and solvents were obtained from commercial sources and used as supplied.

Crude products of the syntheses were purified by liquid column chromatography using Kieselgel 60 (230-400 mesh) (Macherey-Nagel). The progress of the reactions was monitored using analytical thin-layer chromatography (TLC) using aluminum-coated sheets with silica gel with fluorescent indicator F_{254} (Merck, Darmstadt, Germany). The compounds on TLC plates were visualized by sprayed with a solution of 1% cerium(IV) sulfate and 2% phosphomolybdic acid in 5% sulfuric acid and heated or visualized with UV light (λ = 254 nm).

The structures of the synthesized compounds were characterized using nuclear magnetic resonance (NMR). All spectra were measured on a Bruker Avance III HD 500 MHz (500 MHz for ^{1}H NMR, 126 for ^{13}C NMR) spectrometer (Bruker, Billerica, MA, USA). Compounds analysis were dissolved in chloroform-*d* and chemical shifts were expressed as parts per million (ppm) relative to internal standard—tetramethylsilane (TMS).

Infrared spectra (FT-IR) were determined using a Tensor 27 FTIR spectrometer (Bruker, Billerica, MA, USA) with an ATR accessory with a diamond crystal in the wavelength range 400–4000 cm^{-1}.

HR ESI-MS (High-resolution electrospray ionization mass spectrometry) spectra were measured on a Bruker ESI-Q-TOF Maxis Impact Mass Spectrometer (Bruker, Billerica, MA, USA). Ionization mass spectra were collected at the ranges of m/z 50–1300 in a positive ion mode. The samples were dissolved in 1.0 mL of acetonitrile and diluted 1000-fold with acetonitrile containing 0.1% of formic acid. For calibration, the solution with sodium formate clusters in the mass range of 50–1300 m/z was used.

The melting point temperatures were measured using a polarized optical microscope, DM2700P (Leica Microsystems GmbH, Wetzlar, Germany), equipped with a heating/cooling stage LTS420 (Linkam Scientific Instruments Ltd., Salfords, UK). The samples were prepared between two microscopic slides, placed on the Peltier stage of the heating/cooling stage, and the changes were observed under a microscope. The melting temperatures were determined in the heating cycle at a heating rate of 10 °C/min.

S2. Synthesis of 1-(6-Dimethylaminonaphthalen-2-yl)ethanone (ADN1) [1]

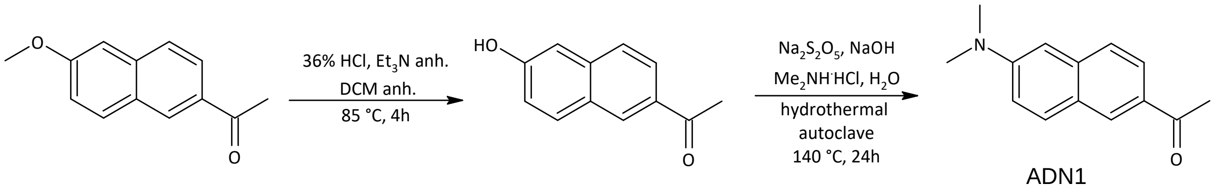

Scheme S1. Synthesis of ADN1.

1-(6-Methoxy-2-naphthalenyl)ethanone (0.750 g, 2.50 mmol) was dissolved in 6 mL of anhydrous dichloromethane. Then the mixture was added dropwise into a round-bottom flask containing 36% HCl (50 mL, 0.465 mol) with constant stirring. Next, anhydrous triethylamine (0.50 mL, 2.70 mmol) was added and the reaction was heated at 85 °C under reflux for 4 h. The excess HCl was neutralized with solid NaOH. The reaction mixture was transferred to a separatory funnel and extracted with ethyl acetate (4x80 mL). The collected organic layers were dried over anhydrous Na_2SO_4. The mixture was filtered and the solvent was evaporated on a vacuum evaporator. The crude product was purified on column chromatography using silica gel as a stationary phase and a mixture of *n*-hexane:ethyl acetate 5:3 (v/v) as eluant to obtain a pure product as an off-white solid (0.556 g, 79.7%).

Step II

1-(6-Hydroxy-2-naphthyl)ethanone obtained in Step I (0.475 g, 1.78 mmol), $Na_2S_2O_5$ (0.834 g, 3.56 mmol), NaOH (0.468 g, 8.93 mmol), $Me_2NH·HCl$ (0.598 g, 7.98 mmol) and 15 mL of deionized water were mixed in hydrothermal autoclave. The reaction was heated at 140 °C for 48 hours. The solid residue was then filtered off and washed with deionized water until pH 7. The crude product was purified on column chromatography using silica gel as a stationary phase and a mixture of *n*-hexane:ethyl acetate 5:1 (v/v) as eluent to obtain a pure product as a light yellow solid (0.155 g, 28.5%).

1-(6-Dimethylaminonaphthalen-2-yl)ethanone (ADN1)

light yellow solid, 0.155g (28.6% yield), m.p. 153-156 °C;
^{1}H NMR (500 MHz, $CDCl_3$) δ 8.32 (d, *J* = 1.8 Hz, 1H), 7.92 (dd, *J* = 8.7, 1.9 Hz, 1H), 7.80 (d, *J* = 9.1 Hz, 1H), 7.63 (d, *J* = 8.7 Hz, 1H), 7.17 (dd, *J* = 9.1, 2.6 Hz, 1H), 6.87 (d, *J* = 2.6 Hz, 1H), 3.11 (s, 6H), 2.67 (s, 3H); ^{13}C NMR (126 MHz, CDCl3) δ 197.84, 150.41, 137.82, 130.97, 130.83, 130.50, 126.31, 125.19, 124.74, 116.41, 105.47, 40.56, 26.54; FTIR-ATR [cm^{-1}]:

2902.76, 1611.93, 1660.88, 1508.22, 1446.45, 1383.89, 1356.29, 1289, 1219.71, 1186.4, 1066.92, 963.19, 938.88, 95.16, 853.41, 805.43, 671.84, 600.5, 543.83; HR ESI-MS *m/z* calculated for $C_{14}H_{16}NO$ $[M+H]^+$ 214.1226, found $[M+H]^+$ 214.1232. 472,77.

S3. Synthesis of (*E*)-4-(6-(Dimethylamino)naphthalen-2-yl)but-3-en-2-one (ADN2) [2]

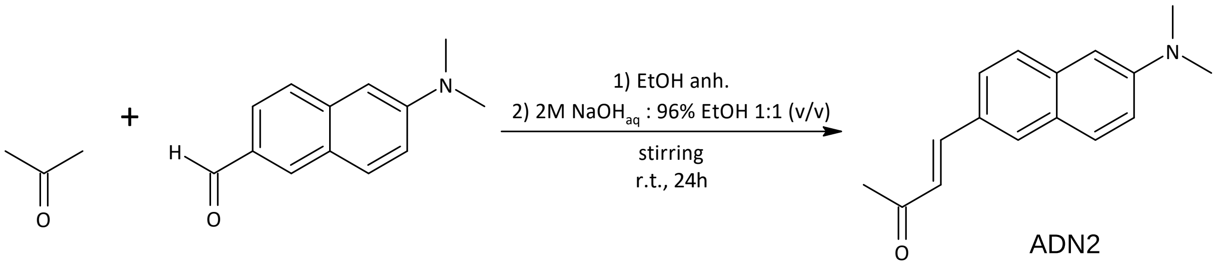

Scheme S2. Synthesis of ADN1.

Acetone (147 µL, 2 mmol) and 6-dimethylamino-2-naphthaldehyde (199 mg, 1 mmol) were dissolved in 12 mL of anhydrous ethanol on a magnetic stirrer. Then a 1:1 (v/v) mixture of 2M NaOH and 96% ethanol (24 mL) was added dropwise. The reaction was continued at room temperature for 24 h, after which the solvent was evaporated on a vacuum evaporator. The residue was transferred to a separatory funnel and deionized water (100 mL) was added. The aqueous layer was extracted with dichloromethane (4x50 mL). The collected organic layers were dried over anhydrous Na_2SO_4. The mixture was filtered and the solvent was evaporated on a vacuum evaporator. The crude product was purified on column chromatography using silica gel as a stationary phase and a mixture *n*-hexane:ethyl acetate 1:1 (v/v) as eluent to obtain pure product as yellow solid (77.0 mg, 32.2%).

(*E*)-4-(6-(Dimethylamino)naphthalen-2-yl)but-3-en-2-one (ADN2)

yellow solid, 77.0 mg (32.2% yield), m.p. 139-142 °C;

^{1}H NMR (500 MHz, $CDCl_3$) δ 7.80 (d, *J* = 1.7 Hz, 1H), 7.71 (dd, *J* = 9.0, 0.7 Hz, 1H), 7.67 – 7.59 (m, 2H), 7.57 (dd, *J* = 8.6, 1.8 Hz, 1H), 7.14 (dd, *J* = 9.1, 2.6 Hz, 1H), 6.87 (d, *J* = 2.5 Hz, 1H), 6.74 (d, *J* = 16.2 Hz, 1H), 3.09 (s, 6H), 2.39 (s, 3H); ^{13}C NMR (126 MHz, $CDCl_3$) δ 198.59, 149.75, 144.52, 136.48, 130.68, 129.81, 128.03, 127.03, 126.22, 125.18, 124.18, 116.50, 106.01, 40.66, 27.56; FTIR-ATR [cm^{-1}]: 2886.61, 1626.13, 1592.86, 1505.43, 1384.31, 1353.7, 1241.71, 1170.53, 970.37, 898.99, 834.85, 806.62, 666.14, 564.12, 475.69; HR ESI-MS *m/z* calculated for $C_{16}H_{18}NO$ $[M+H]^+$ 240.1383, found $[M+H]^+$ 240.1390.

S4. Synthesis of (*E*)-3-(6-(Dimethylamino)naphthalen-2-yl)-1-phenylprop-2-en-1-one (ADN3) [2]

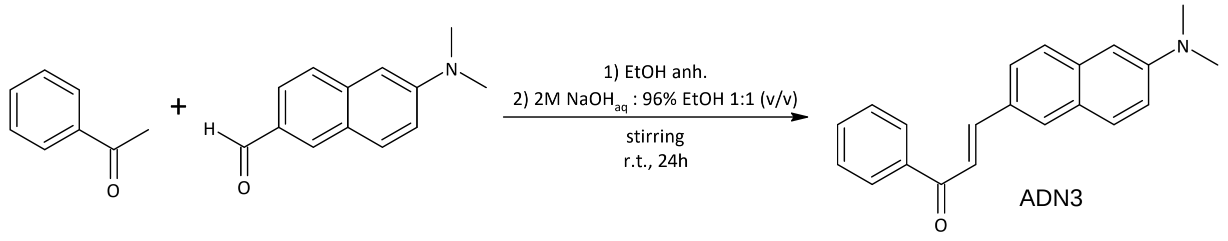

Scheme S3. Synthesis of ADN3.

Acetophenone (175 µL, 1.5 mmol) and 6-dimethylamino-2-naphthaldehyde (199 mg, 1 mmol) were dissolved in 12 mL of anhydrous ethanol on a magnetic stirrer. Then a 1:1 (v/v) mixture of 2M NaOH and 96% ethanol (24 mL) was added dropwise. The reaction was continued at room temperature for 24 h, after which the solvent was evaporated on a vacuum evaporator. The residue was transferred to a separatory funnel and deionized water (100 mL) was added. The aqueous layer was extracted with dichloromethane (4x50 mL). The collected organic layers were dried over anhydrous Na_2SO_4. The mixture was filtered and the solvent was evaporated on a vacuum evaporator. The crude product was purified on column chromatography using silica gel as a stationary phase and a mixture *n*-hexane:ethyl acetate 5:2 (v/v) as eluent to obtain pure product as orange solid (253 mg, 84.0%).

(*E*)-3-(6-(Dimethylamino)naphthalen-2-yl)-1-phenylprop-2-en-1-one (ADN3)

Orange solid, 253 mg (84.0% yield), m.p. 135-138 °C

^{1}H NMR (500 MHz, $CDCl_3$) δ 8.08 – 8.02 (m, 2H), 7.95 (d, *J* = 15.7 Hz, 1H), 7.88 (d, *J* = 1.7 Hz, 1H), 7.74 (d, *J* = 9.1 Hz, 1H), 7.70 (dd, *J* = 8.7, 1.8 Hz, 1H), 7.65 (d, *J* = 8.6 Hz, 1H), 7.61 – 7.49 (m, 4H), 7.15 (dd, *J* = 9.1, 2.6 Hz, 1H), 6.89 (d, *J* = 2.5 Hz, 1H), 3.10 (s, 6H); ^{13}C NMR (126 MHz, $CDCl_3$) δ 190.81, 149.79, 146.00, 138.91, 136.57, 132.58, 131.15, 129.94, 128.69, 128.59, 128.57, 126.97, 126.28, 124.36, 119.95, 116.48, 106.04, 40.66; FTIR-ATR [cm^{-1}]: 2907,33, 1650,52, 1623,67, 1559,16, 1423,84, 1301,64, 1261,26, 1204,49, 1171,94, 984,33, 895,68, 843,95, 804,63, 780,7, 695,84, 653,59, 593,31, 474,78; HR ESI-MS *m*/*z* calculated for $C_{21}H_{20}NO$ [M+H]$^+$ 302.1548, found [M+H]$^+$ 240.1390.

References:

1. Yao, W.; Yan, Y.; Xue, L.; Zhang, C.; Li, G.; Zheng, Q.; Zhao, Y.S.; Jiang, H.; Yao, J. Controlling the Structures and Photonic Properties of Organic Nanomaterials by Molecular Design. *Angew. Chemie Int. Ed.* **2013**, *52*, 8713–8717, doi:10.1002/anie.201302894.
2. Koo, J.Y.; Heo, C.H.; Shin, Y.H.; Kim, D.; Lim, C.S.; Cho, B.R.; Kim, H.M.; Park, S.B. Readily Accessible and Predictable Naphthalene-Based Two-Photon Fluorophore with Full Visible-Color Coverage. *Chem. - A Eur. J.* **2016**, *22*, 14166–14170, doi:10.1002/chem.201603496.

S5. Spectra of the synthesized compounds

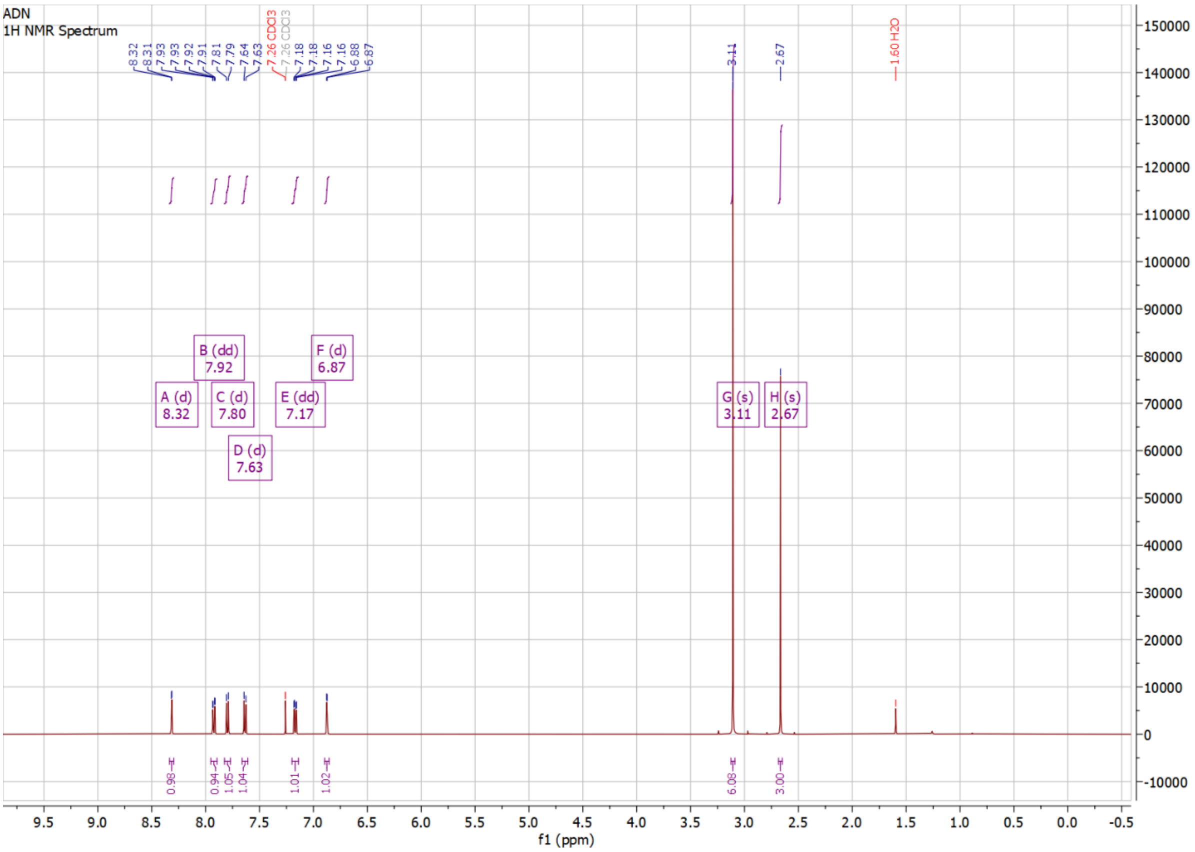

Figure S1. ^{1}H-NMR (500 MHz, $CDCl_3$) spectrum of 1-(6-Dimethylaminonaphthalen-2-yl)ethanone (ADN1).

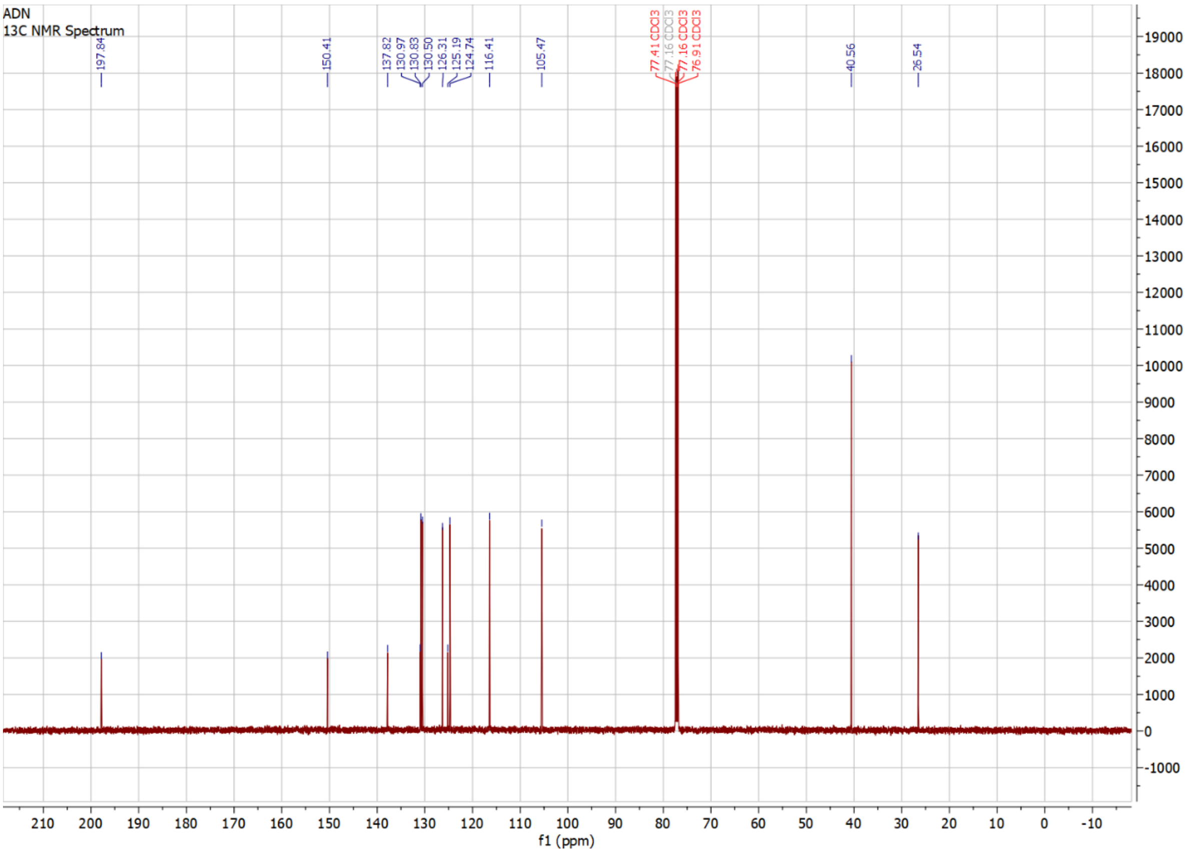

Figure S2. ^{13}C-NMR (126 MHz, $CDCl_3$) spectrum of 1-(6-Dimethylaminonaphthalen-2-yl)ethanone (ADN1).

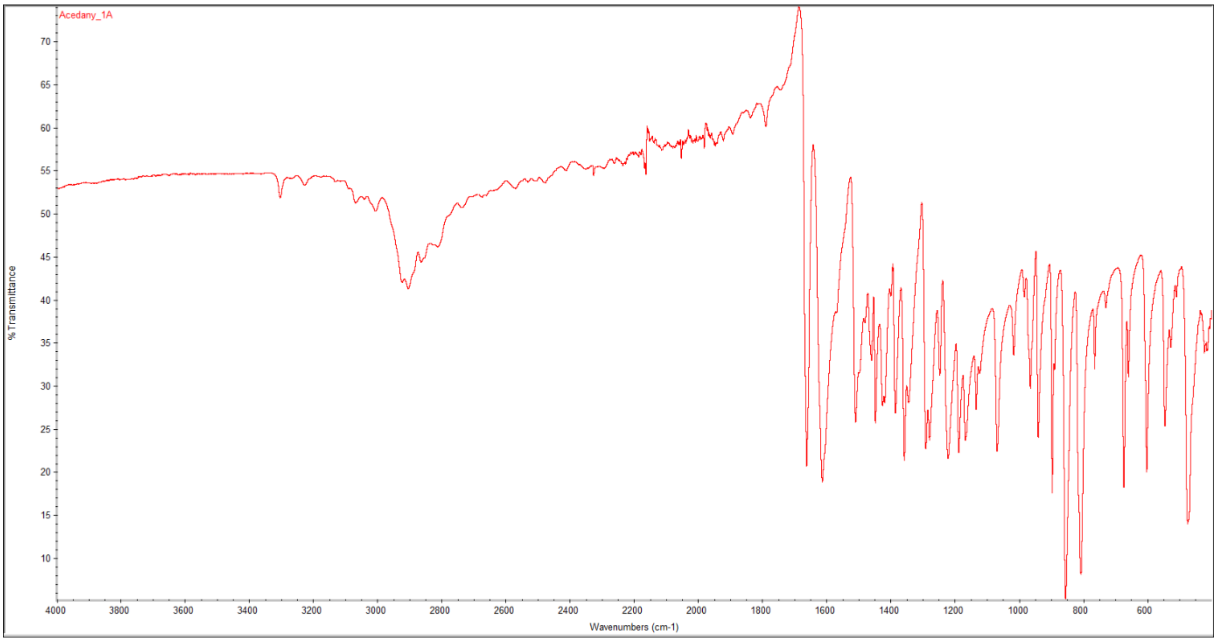

Figure S3. FT-IR (ATR) spectrum of 1-(6-Dimethylaminonaphthalen-2-yl)ethanone (ADN1).

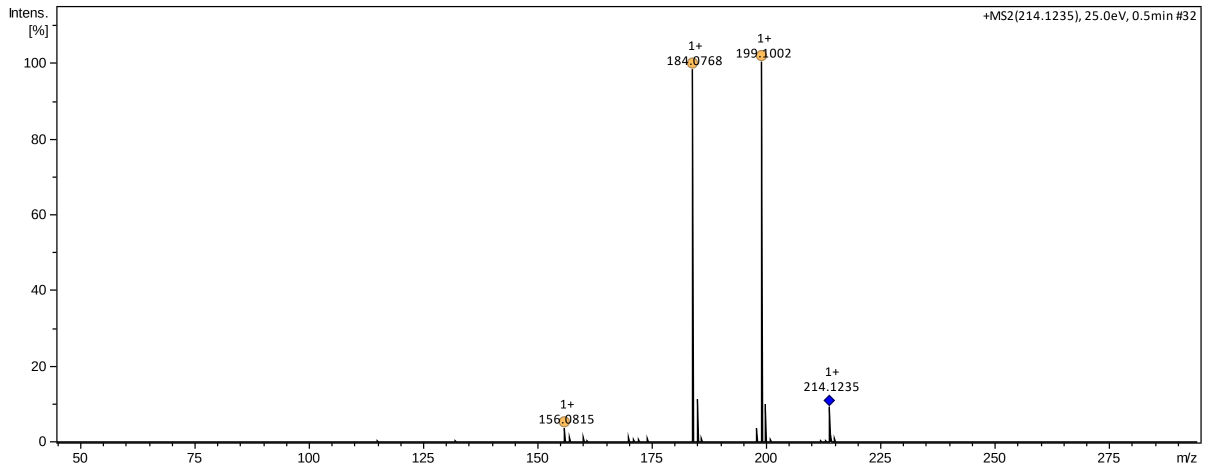

Figure S4. HR ESI-MS spectrum of 1-(6-Dimethylaminonaphthalen-2-yl)ethanone (ADN1).

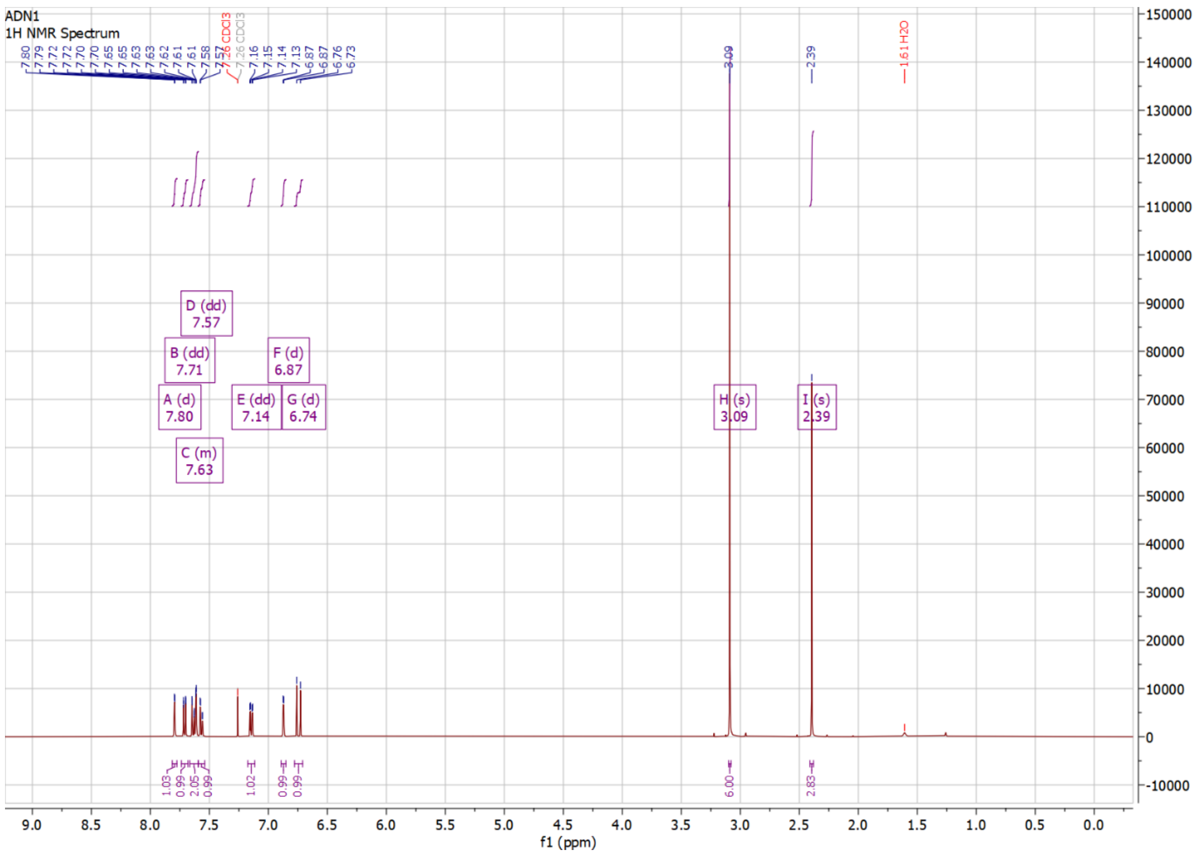

Figure S5. ^{1}H-NMR (500 MHz, $CDCl_3$) spectrum of (*E*)-4-(6-(Dimethylamino)naphthalen-2-yl)but-3-en-2-one (ADN2).

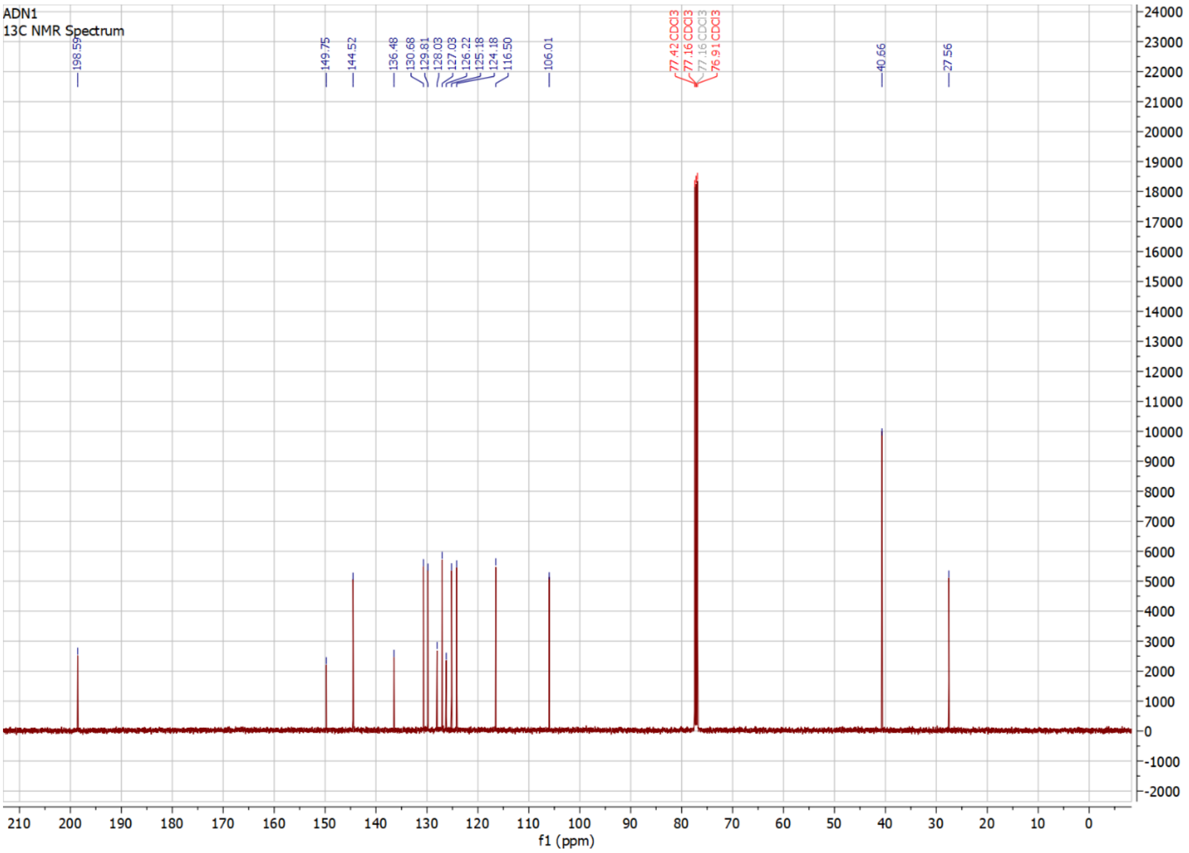

Figure S6. ^{13}C-NMR (126 MHz, $CDCl_3$) spectrum of (*E*)-4-(6-(Dimethylamino)naphthalen-2-yl)but-3-en-2-one (ADN2).

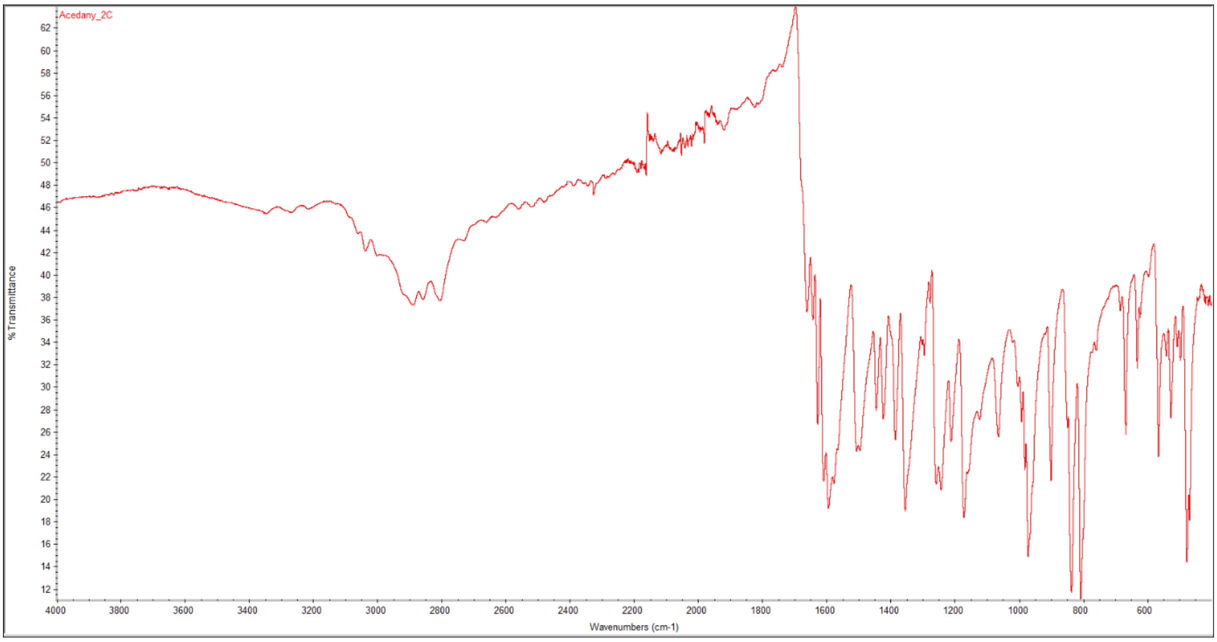

Figure S7. FT-IR (ATR) spectrum of (*E*)-4-(6-(Dimethylamino)naphthalen-2-yl)but-3-en-2-one (ADN2).

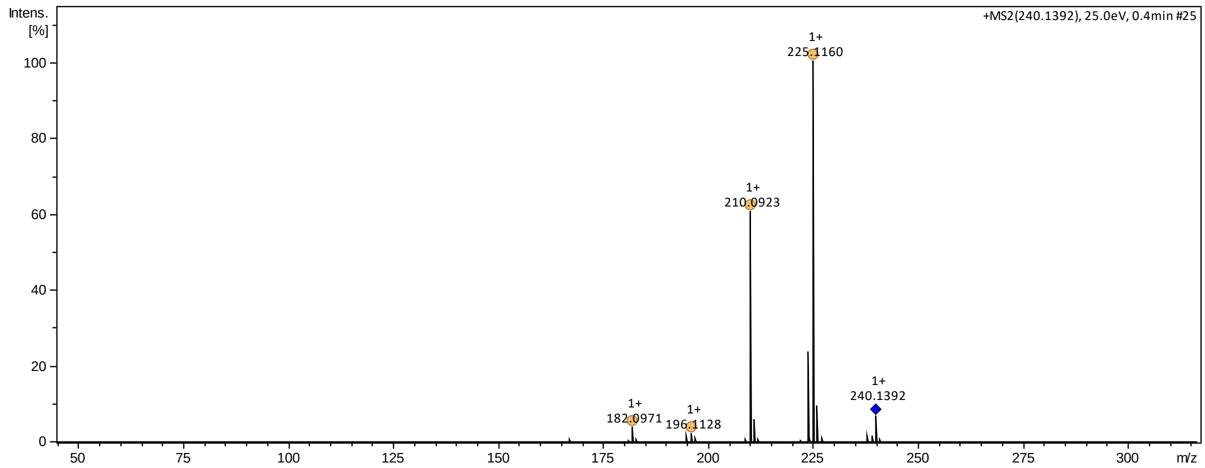

Figure S8. HR ESI-MS spectrum of (*E*)-4-(6-(Dimethylamino)naphthalen-2-yl)but-3-en-2-one (ADN2).

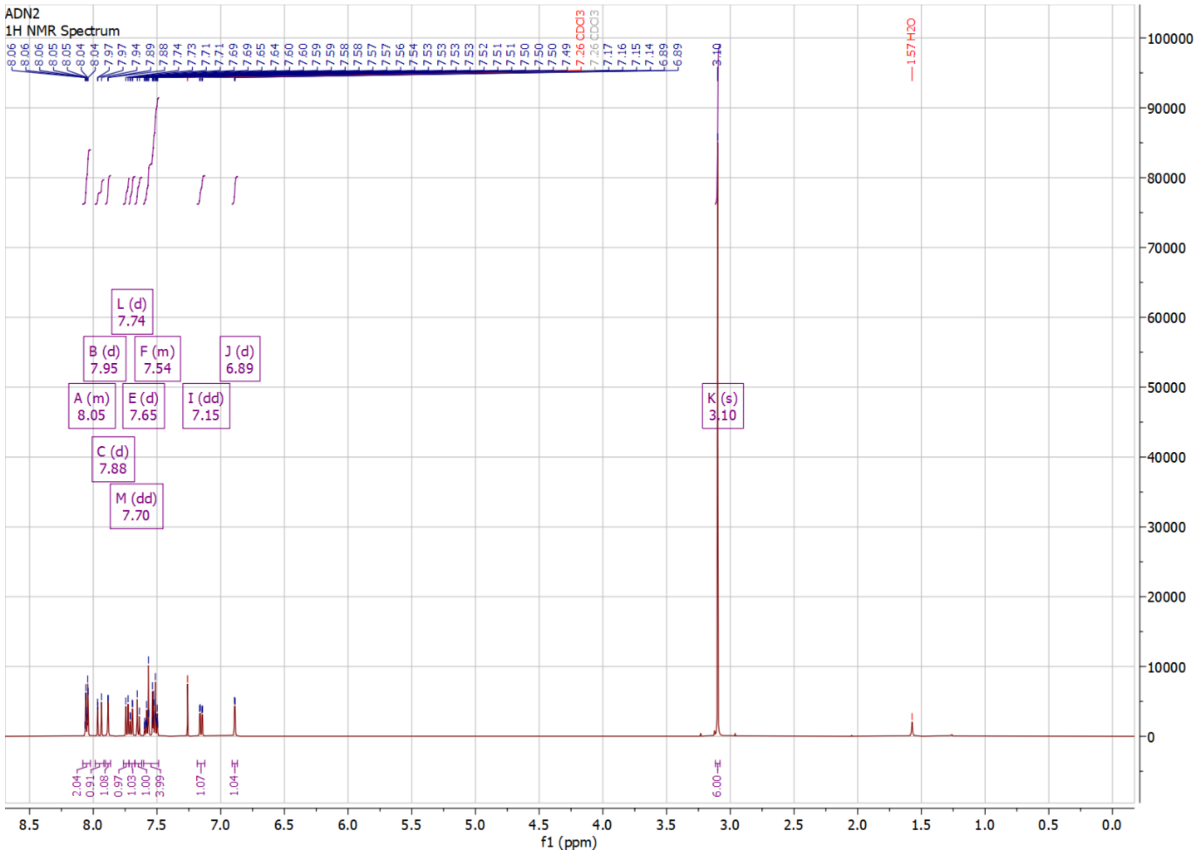

Figure S9. ^{1}H-NMR (500 MHz, $CDCl_3$) spectrum of (*E*)-3-(6-(Dimethylamino)naphthalen-2-yl)-1-phenylprop-2-en-1-one (ADN3).

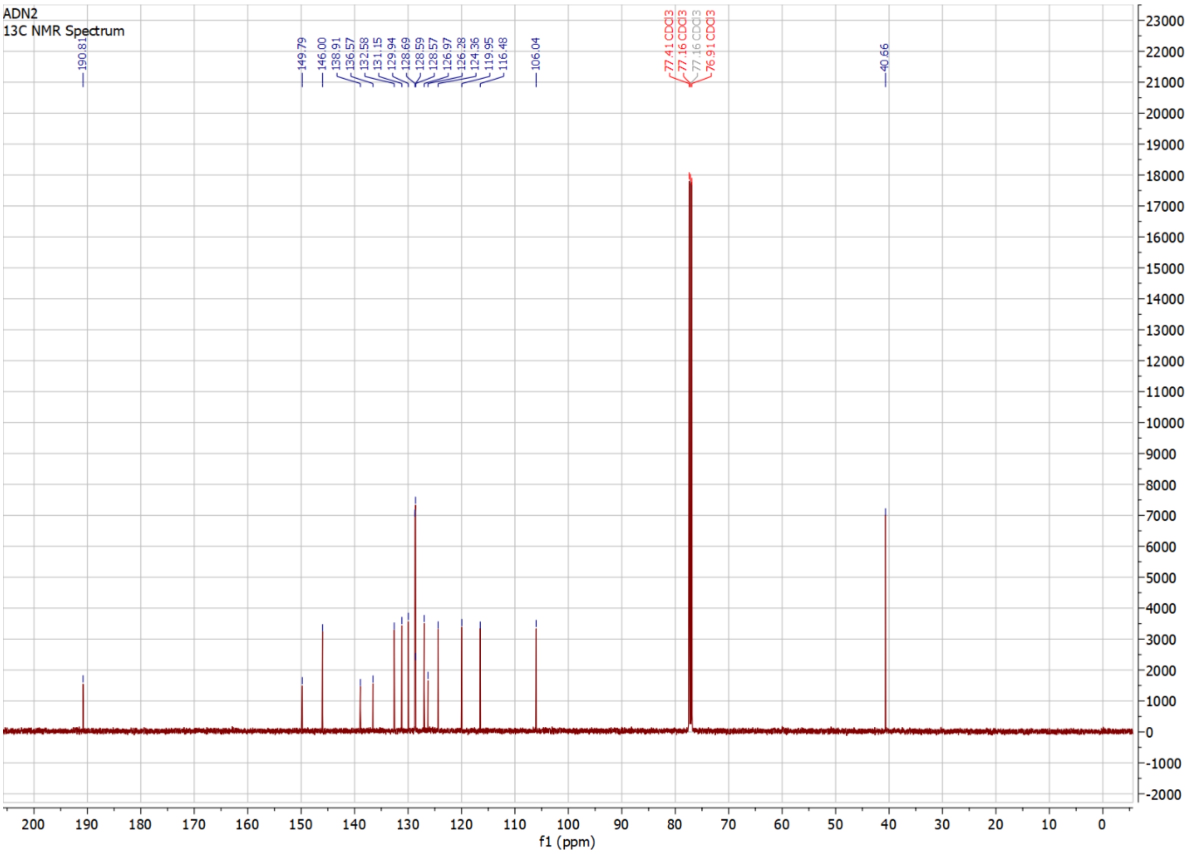

Figure S10. ^{13}C-NMR (126 MHz, $CDCl_3$) spectrum of (*E*)-3-(6-(Dimethylamino)naphthalen-2-yl)-1-phenylprop-2-en-1-one (ADN3).

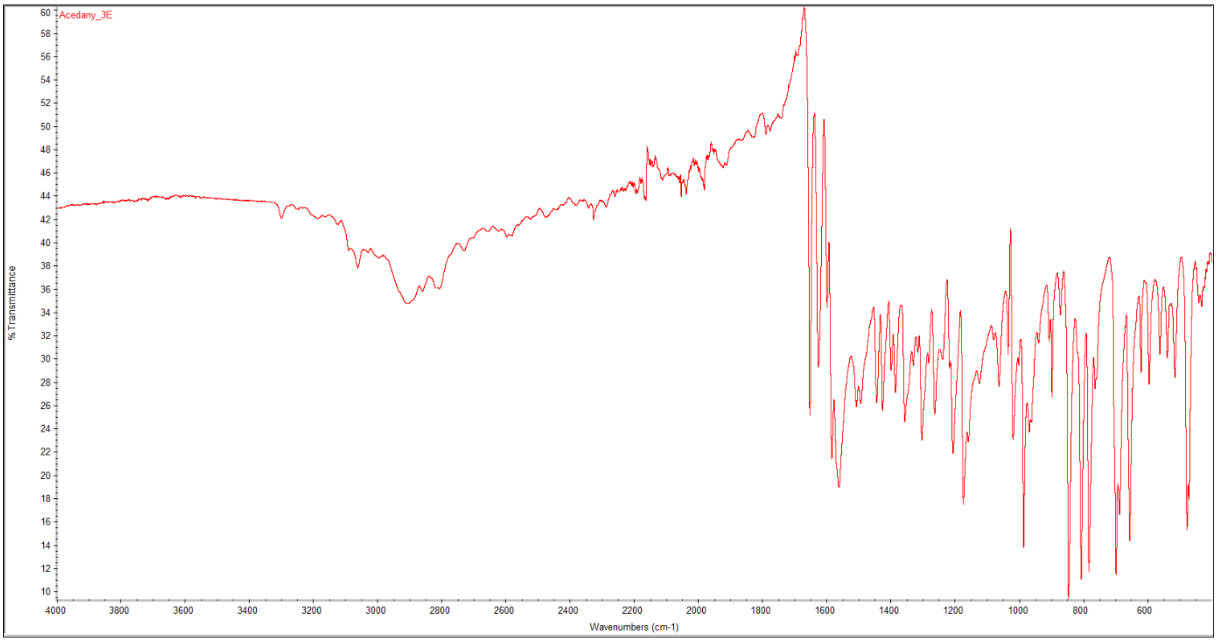

Figure S11. FT-IR (ATR) spectrum of (*E*)-3-(6-(Dimethylamino)naphthalen-2-yl)-1-phenylprop-2-en-1-one (ADN3).

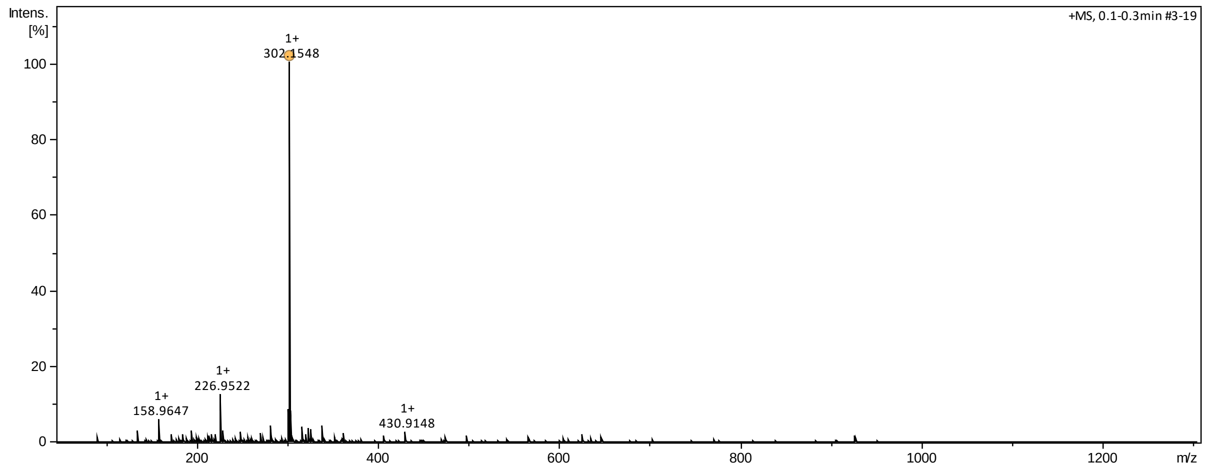

Figure S12. HR ESI-MS spectrum of (*E*)-3-(6-(Dimethylamino)naphthalen-2-yl)-1-phenylprop-2-en-1-one (ADN3).

S3. THG response of the glass plate.

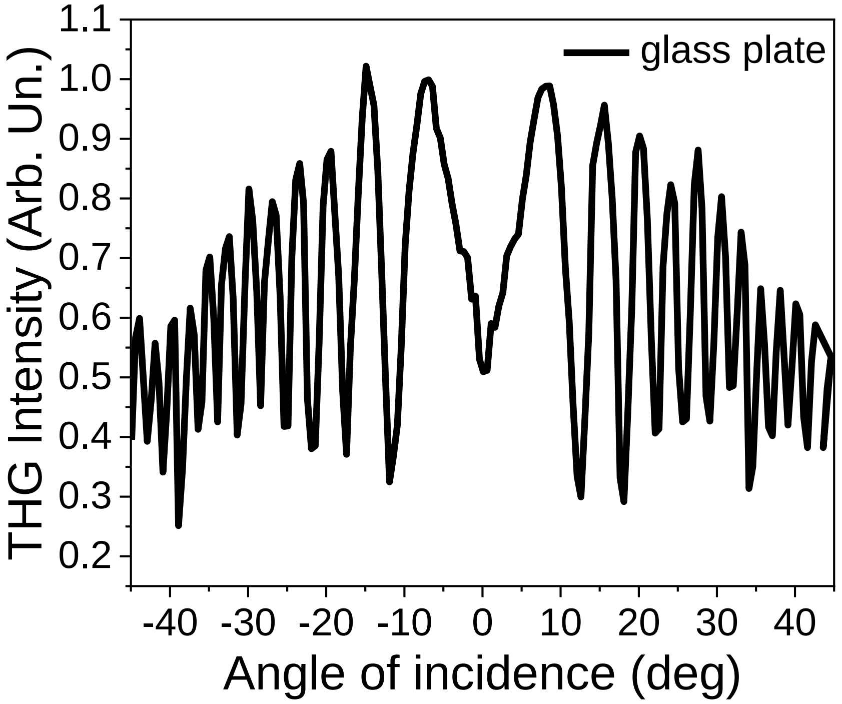

Figure S13. The Maker Fringes pattern of the glass plate.

S4. The basic spectroscopic properties of thin films containing ADN1, ADN2, and ADN3 in the PMMA matrix.

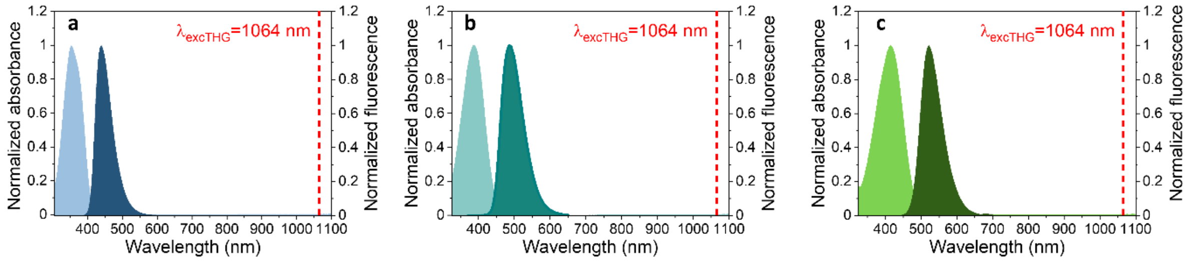

Figure S14. The absorbance and fluorescence spectra of AND1 (a), ADN2 (b), and ADN3 (c) in PMMA.

Compound ADN1 (S3a) exhibits light absorption in the range of 300 - 425 nm, with a maximum of 352 nm. Its fluorescence spectrum covers a rather wide range from 400 to 550 nm, with a λ_{max} at 440 nm, falling within the blue region of the visible light. ADN2 (S3b), on the other hand, displays an absorption range spanning from 300 to 475 nm, with a prominently visible maximum at 386.5 nm. Fluorescence spectra are recorded within the range of 425 - 600 nm, with a peak at 486 nm, showing a redshift of 46 nm in the emission wavelength, when compared to ADN1. The emission color can be classified as turquoise. The third dye, ADN3 (S3c) shows very wide absorption ranging from 325 up to 500 nm, with a maximum at 415 nm. The respective fluorescence spectrum spans from 450 nm to 625 nm, with a peak at 520 nm, displaying the classic green emission color. The maximum exhibits an 80 nm redshift compared to ADN1. Considering optical transparency, none of the dyes in our systems absorb in the spectral range of pumping wavelength used in THG studies (λ_{exc} = 1064 nm). As there is no resonance, and the samples are optically transparent, the probability of photodegradation under these conditions is negligible. In the study by Kulyk et al. [3], THG measurements were conducted at an energy level of 150 μJ for an organic dye system in a PMMA host. As shown in their publication, the logarithmic plots of the THG intensity dependence on laser intensity have a linear slope of approximately 2.9, confirming a pure THG process and the absence of damage in the investigated films under the applied laser intensities. In our study, we utilized a laser energy of 100 μJ, which is lower than the value used by Kulyk et al.

3. Kulyk, B.; Guichaoua D.; Ayadi, A.; El-Ghayoury, A.; Sahraoui, B. Functionalized azo-based iminopyridine rhenium complexes for nonlinear optical performance, *Dyes and Pigments*, **2017**, *145*, 256-262. https://doi.org/10.1016/j.dyepig.2017.06.012

S5. The dyes' absorbance and fluorescence spectra demonstrate the spectral overlapping for FRET analysis

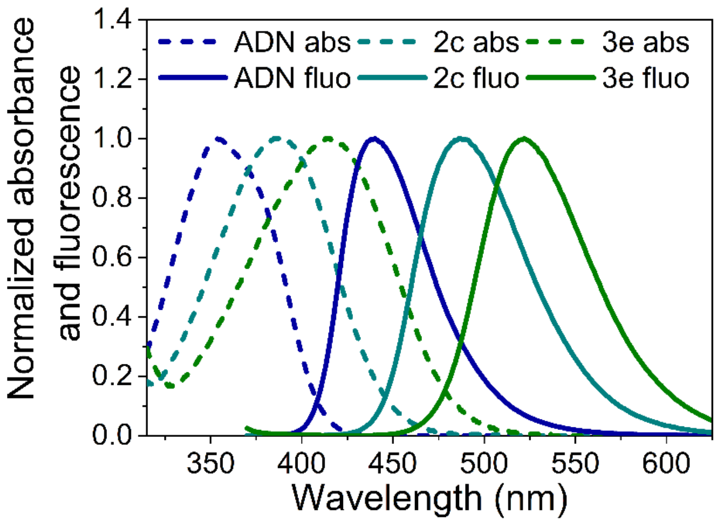

Figure S15. Absorbance and fluorescence of single dyes, demonstrating the spectral overlapping.

S6. The fluorescence lifetime decays of single ADN1, ADN1 in double, and three-dyes arrangements in PMMA.

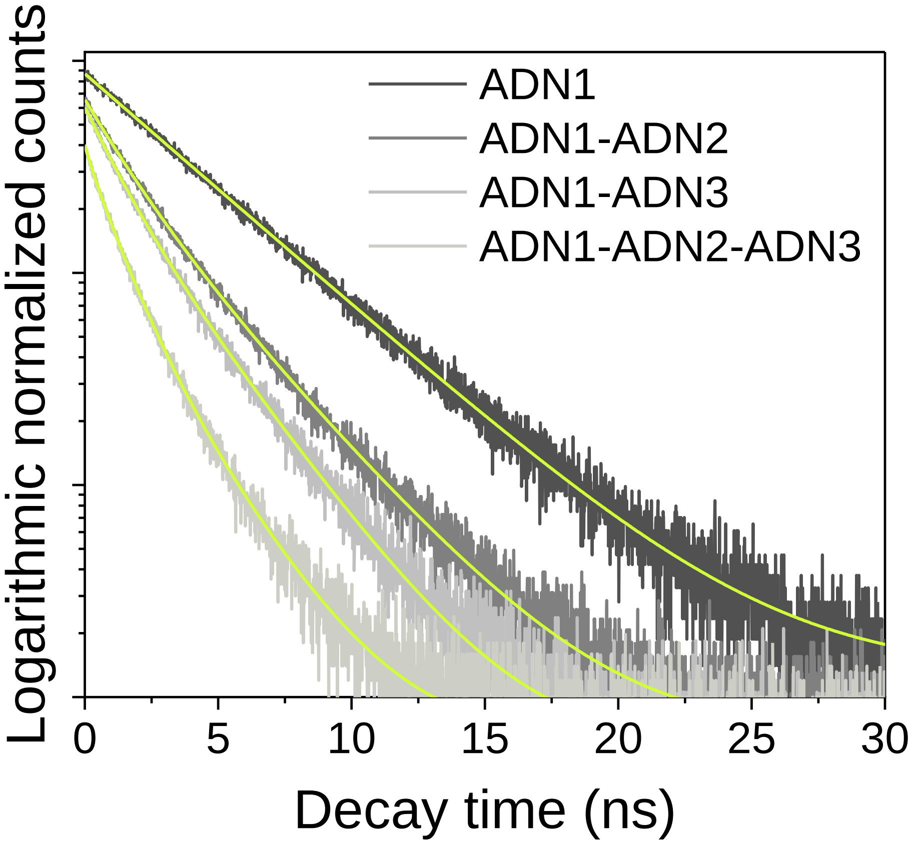

Figure S16. Fluorescence decay curves in single-dye system and investigated mixtures containing ADN1, ADN2, and ADN3 in PMMA.

The software provided by Edinburgh Instruments was used to calculate luminescence decay times as well as Origin Pro software. In Origin Pro software "ExpDec1" model was used for ADN layer, resulting in equation: $y = A1 \cdot e^{-\frac{x}{t1}} + y_0$, where t_1 is luminescence decay time and "ExpDec2" for layers containing two components with equation: $y = A1 \cdot e^{-\frac{x}{t1}} + A2 \cdot e^{-\frac{x}{t2}} + y_0$,, while "ExpDec3" model for three components mixture, using equation: $y = A1 \cdot e^{-\frac{x}{t1}} + A2 \cdot e^{-\frac{x}{t2}} + A3 \cdot e^{-\frac{x}{t3}} y_0$. In case of not single exponential lifetimes – average luminescence lifetime was calculated by: $\tau_{ave} = \sum_{i=1}^{n} Ai\ \tau i$.
